# Identification and functional analysis of sRNAs in *Pseudomonas aeruginosa* quorum sensing and virulence regulation

**DOI:** 10.1016/j.gendis.2026.102292

**Published:** 2026-06-11

**Authors:** Tianmin Li, Beifang Lu, Fang Chen, Letong Xu, Yizhou Zhang, Fangzhou Xie, Yue Sun, Chunyan Yao, Jiadai Huang, Tianlong He, Youyue Li, Yiqing Ding, Haiyan Hu, Shaojun Tang, Xin Deng

**Affiliations:** a Department of Biomedical Sciences, City University of Hong Kong, Kowloon Tong, Hong Kong SAR 999077, China; b Bioscience and Biomedical Engineering Thrust, System Hub, The Hong Kong University of Science and Technology (Guangzhou), Guangzhou, Guangdong 511466, China; c Division of Life Science, The Hong Kong University of Science and Technology, Clear Water Bay, Hong Kong SAR 999077, China; d Institute of Fruit Tree Research, Guangdong Academy of Agricultural Sciences, Key Laboratory of South Subtropical Fruit Biology and Genetic Resource Utilization, Ministry of Agriculture and Rural Affairs, Guangdong Provincial Key Laboratory of Science and Technology Research on Fruit Tree, Guangzhou, Guangdong 510640, China; e School of Pharmaceutical Sciences, Sun Yat-Sen University, University Town, Guangzhou, Guangdong 510006, China; f Department of Biostatistics, Virginia Commonwealth University, Richmond, VA 23219, USA; g Massey Comprehensive Cancer Center, Virginia Commonwealth University, Richmond, VA 23219, USA; h Shenzhen Research Institute, City University of Hong Kong, Shenzhen, Guangdong 518057, China

**Keywords:** Pan-sRNome, *Pseudomonas aeruginosa*, Quorum sensing, Regulatory network, Small RNA

## Abstract

Small RNAs (sRNAs) act as critical regulators of post-transcriptional activity and diverse cellular processes in bacteria. However, the entire repertoire of sRNAs and their functions has not yet been extensively studied. This study employs a novel combined computational-experimental prediction approach to identify and characterize small RNAs (sRNAs) in *Pseudomonas aeruginosa* PAO1. We defined the first pan-sRNome, including 1663 sRNAs across 484 *P. aeruginosa* strains, and classified our 9 newly discovered sRNAs as 3 core and 6 accessory elements within the regulatory network. Functional studies revealed distinct sRNA activities: PA0982.1 was found to be a direct regulator of the *rhl* quorum sensing system. PA1014.1, PA2734.1, and PA2736.2 enhanced pyocyanin production. PA1014.1 and PA2734.1 facilitated biofilm formation for surface attachment. PA2734.1 and PA2736.2 suppressed motility to regulate dispersal, collectively shaping virulence traits. Additionally, PA1270.1, PA1367.1, and PA3471.1 were associated with reduced interbacterial competitiveness and the downregulation of the Type VI secretion system. Evolutionary analysis across diverse bacterial taxa and archaea demonstrated selective conservation, exposing the evolutionary trajectory of a diverse group of regulatory molecules. Overall, this study not only expands the *P. aeruginosa* sRNA landscape but also provides a framework for understanding how sRNAs fine-tune virulence and evolutionary adaptation.

## Introduction

Small RNAs (sRNAs) have been found in all domains of life and play important roles in the regulation of gene expression, influencing transcriptional and translational processes.^1^ Landmark discoveries in recent decades, such as the identification of *lin-4* and *let-7* in *Caenorhabditis elegans*, have revolutionized our understanding of the central dogma and revealed a diverse world of regulatory non-coding RNAs.^2^ In eukaryotes, these are categorized into distinct biological classes based on their biogenesis and function. MicroRNAs (miRNAs) act as post-transcriptional repressors by guiding the Argonaute protein to target mRNAs. In parallel, small interfering RNAs (siRNAs) mediate RNA interference (RNAi) to defend against foreign genetic material. Additionally, PIWI-interacting RNAs (piRNAs) are crucial for silencing transposons to maintain germline genome stability.^3^ Furthermore, recent studies have highlighted non-canonical classes such as transfer RNA (tRNA)-derived small RNAs (tsRNAs), which are generated through specific cleavage of tRNAs and regulate processes including translation and stress responses.^4^ While these eukaryotic mechanisms are well characterized, bacterial sRNAs typically range from 50 to 500 nucleotides in length and exert regulatory functions.^5^ Historically, sRNAs have been identified through systematic searches based on criteria such as sequence conservation, the presence of orphan promoters and terminators, or copurification with proteins.^6^, ^7^, ^8^ Following the development of high-throughput sequencing and bioinformatics analyses, genome-wide identification of non-coding RNA (ncRNA) molecules has revealed a vast repertoire of sRNAs.^9^ Consequently, several computational methods for predicting bacterial sRNA-encoding genes have recently emerged. In well-studied bacteria such as *Escherichia coli* (*E. coli*) and *Salmonella enterica* (*S. enterica*), over 300 sRNAs have been annotated.^10^ A regulatory RNA database has cataloged up to 159 putative sRNAs in *Staphylococcus aureus* (*S. aureus*),^11^ with approximately 20 experimentally verified sRNAs.^12^^,^^13^ Similarly, over 100 sRNAs have been identified in *Listeria monocytogenes*, of which approximately 30 have been experimentally validated.^14^^,^^15^ However, functional characterization of sRNAs in prokaryotes has posed significant challenges. The expression of certain sRNAs is condition dependent, occurring in response to specific environmental conditions such as low iron availability, low oxygen levels, or within the host environment.^7^ In addition, sRNAs in bacteria often exhibit functional redundancy. For instance, in *Pseudomonas aeruginosa*, the sRNAs RsmY and RsmZ function redundantly, such that the loss of one sRNA can be compensated by the other.^16^ In these cases, single-gene deletions may yield no observable phenotype, whereas overexpression can effectively uncover their regulatory potential and physiological capacity. This approach is widely accepted as a strategy for elucidating sRNA regulation when the inducing conditions remain unknown.^17^

sRNAs often base pair with their target mRNAs through short complementary sequences. These interactions can inhibit translation initiation by blocking access to the ribosome-binding site or can promote mRNA decay by recruiting ribonucleases, thereby leading to down-regulation of gene expression at the post-transcriptional level.^18^ For instance, the sRNA MicC in *E. coli* inhibits the translation of ompC mRNA through base pairing and promotes its degradation.^19^ Mechanistically, bacterial sRNAs may regulate target genes by several distinct modes of action. They can (i) base-pair with complementary mRNAs to promote degradation by RNases,^20^ which usually involves the RNA chaperone Hfq.^17^^,^^21^ Alternatively, they can (ii) bind to target mRNAs to form RNA–RNA duplexes that inhibit or terminate translation.^22^ Furthermore, sRNAs can positively regulate targets by (iii) interacting with regulatory proteins and modulating their activity.^23^ Finally, they can (iv) base-pair to the 3′ or 5′-untranslated region (UTR) to enhance mRNA stability or promote translation.^24^^,^^25^

Studies investigating sRNAs in model organisms such as *E. coli*, *S. enterica*, and *S. aureus* have elucidated the diverse functions of sRNAs.^26^, ^27^, ^28^ For instance, in *E. coli*, the sRNA RyhB is involved in iron homeostasis by regulating genes associated with iron uptake and storage.^29^ In *S. enterica*, the sRNA MicA was shown to play a crucial role in biofilm formation, thereby enhancing virulence.^30^ In *S. aureus*, the sRNA SprD contributes to virulence by regulating immune evasion factors.^31^ Taken together, these previous findings suggest that exploring the roles of newly identified sRNAs in pathogens would be crucial for understanding their potential impact on virulence. *Pseudomonas aeruginosa* is a bacterial pathogen that causes serious nosocomial infections in humans.^32^ The *P. aeruginosa* PAO1 strain is a model organism for studies of biological characteristics, pathogenic mechanisms, antibiotic resistance, and interactions with hosts.^33^ While early studies established the foundation of sRNA identification in this pathogen,^34^^,^^35^ the functional roles of most sRNAs in *P. aeruginosa* remain undefined. Recent advances have underscored the complexity and clinical relevance of its sRNA regulatory targets. For instance, recent RNA interaction by ligation and sequencing analyses have mapped extensive Hfq-mediated RNA–RNA interaction networks, revealing sRNAs as central integrators of transcriptional regulation that subsequently influence the synthesis of the quorum sensing signal.^36^ Furthermore, novel sRNAs have been increasingly implicated in regulating critical virulence traits, such as motility, cytotoxicity, and antibiotic resistance.^37^

In this study, we used a combined computational-experimental prediction (CCEP) approach to identify and characterize functional sRNAs in *P. aeruginosa*. The CCEP approach integrates computational prediction with experimental validation to enhance the accuracy and efficiency of sRNA identification. Importantly, this methodological advancement not only overcomes the challenges of detecting sRNAs under specific growth conditions but also provides a scalable, universal framework for decoding the non-coding regulatory landscapes of other bacterial pathogens. Building upon this, we established the first comprehensive pan-sRNome, categorizing the sRNA repertoire into core, accessory, and unique elements to trace their evolutionary trajectories. Crucially, we elucidated the mechanism of PA0982.1, demonstrating its role as a direct regulator of the *rhl* QS system. This finding adds a critical layer of post-transcriptional control to bacterial communication. By mapping these sRNAs to specific virulence traits, our work highlights their pivotal roles in fine-tuning the virulence of *P. aeruginosa*.

## Materials and methods

### Bacterial strains, plasmids, and growth conditions

In this study, *P. aeruginosa* PAO1 (Assembly Accession: GCF_000006765.1) served as the wild-type strain. *E. coli* DH5α (Assembly Accession: GCF_002899475.1) was employed as competent cells for recombinant plasmid amplification. The plasmid pUC18, conferring gentamycin (Gm) resistance in *E. coli* DH5α, was used in the competition assay. Both bacterial strains were routinely cultured in Luria–Bertani (LB) medium, which is optimal for *P. aeruginosa* and *E. coli* growth, and maintained at 37 °C.

### RNA extraction, transcriptome sequencing, and analysis

Overnight cultured *P. aeruginosa* was then inoculated at a 1:100 volume ratio into 2 mL of LB for subculturing. The bacterial pellet was then harvested at the exponential growth phase at an OD_600_ of approximately 0.6. Total RNA was extracted from the wild-type (WT) *P. aeruginosa* PAO1 strain using the Direct-zol RNA MiniPrep kit (Zymo Research, Irvine, CA), which is optimized to preserve small sizes ranging from 17 nucleotides (nt) to 200 nt. Total RNA samples were also extracted from the wild-type strain with empty vector (WTEV), sRNA overexpression strains, and Δ*PA0982.1* strain. Following the depletion of ribosomal RNAs (rRNAs), the enriched mRNAs were utilized for complementary DNA (cDNA) library construction. The cDNA libraries were prepared using the RNA Library Prep Kit for Illumina (NEB, England) and sequenced by the HiSeq X Ten platform (Illumina).

Sequencing quality was evaluated by FastQC (v0.12.1), and adapter sequences were removed by Trim Galore (v0.6.10). The processed, high-quality reads were aligned to the PAO1 reference genome using HISAT2 (v2.2.1).^38^ The resulting SAM files were converted to sorted BAM files using SAMtools (v1.9), and gene expression quantification was performed with FeatureCounts (Subread v2.0.1).^39^ The differentially expressed genes (DEGs) were generated by DESeq2 (v1.40.2).^40^

A total of 1443 genes involved in the sRNA regulatory network were filtered with *P*_*adj*_ < 0.001, |log_2_ FC| > 3. Known sRNA interaction networks used the cutoff of *P*_*adj*_ < 0.05, |log_2_ FC| > 1. Both networks were visualized via Cytoscape (v3.10.0).

### Combined computational-experimental prediction (CCEP) for sRNAs

The new sRNA discovery methods were written in the statistical tool R. This workflow requires DNA sequencing depth per base calculated by SAMtools (v1.9) and gft files as inputs. We only included the known ncRNAs when verifying our workflows but not in predicting new ones. ncRNAs are annotated in the third column in the gft file.

Two anchor sequences of at least 30 base pairs (bp) were generated to mitigate systematic bias in RNA processing. A putative sRNA was claimed if the region of interest has a 1.5-fold overall coverage deeper than in surrounding anchor regions. All intergenic regions (IGRs) of PAO1 went through the above signal detection procedures. The visualization of sRNAs was performed with ggplot2 (v3.5.0).^41^

We determined the orientation of predicted sRNAs by calculating the mean scores in corresponding regions from the LM-seq results of PAO1, which were downloaded from GSE175852 and imported in R via rtracklayer (v1.60.1).^42^

### Northern blotting

The bacteria were cultured and collected following the same procedure as described for transcriptome sequencing. Total RNA was extracted using the RNA Isolation Kit (Tiangen, Beijing, China), and 50 μg RNA was loaded into each well of the 6% formaldehyde-2% agarose gel and separated by electrophoresis. The RNA was then transferred via capillary action from the gel to a positively charged nylon membrane (Roche Diagnostics Mannheim, Germany) and cross-linked. To facilitate digoxigenin (DIG)-labeled probes, the transcription process was conducted using an *in vitro* transcription assay with DIG-labeled nucleotides (DIG-11-UTP) according to the DIG Northern Starter Kit (Roche Diagnostics, Mannheim, Germany) instructions. The probe was then applied to the pre-hybridized nylon membrane for subsequent hybridization. Finally, the signal was visualized by immune detection using the Gel Documentation System (Bio-Rad, USA). Each Northern blot was hybridized with a DIG-labeled probe designed to specifically detect a single candidate sRNA. Under our stringent hybridization and washing conditions, signals are only obtained when the target RNA is present, ensuring probe specificity.

### Pan-sRNome analysis

A total of 484 *P. aeruginosa* strain genomes were extracted from NCBI Datasets (https://www.ncbi.nlm.nih.gov/datasets/genome/?taxon=287). These strains are completely assembled, annotated by RefSeq, and released before 2023. A total of 1663 annotated sRNA sequences were retrieved with keywords “sRNA” and “ncRNA” in gff files. Our identified sRNA sequences were incorporated into the *P. aeruginosa* genome annotations and then clustered with a minimum sequence identity of 0.9 and minimum coverage of 0.9 (MMseqs2 Version: 15-6f452).^43^ Fifty-eight representative sRNA sequences were finally used to BLAST in 484 *P. aeruginosa* genome databases. The alignment score is determined by assigning a numerical value to each aligned letter pair and subsequently summing these values over the entire alignment length. We used the threshold *e*-value ≤ 1e-10 and Identity > 90 to perform the final Pan-sRNome study.

### Construction of recombinant plasmids and overexpression strains

The amplified target sRNA gene fragments were cloned and inserted into vector pAK1900 by homologous recombination at the *Bam*HI and *Hind*III restriction sites, which drives high-level transcription of inserted sequences from its promoter in *P. aeruginosa*. The recombinant plasmid was heat-shocked into *E. coli* DH5α competent cells and cultured in LB supplemented with 60 μg/mL carbenicillin (Cb) for plasmid amplification. The amplified plasmids were then extracted and electroporated into PAO1 competent cells, and the empty vector strain served as a control. The overexpression of genes was quantified by reverse transcription quantitative PCR (RT‒qPCR), with a fold change in expression of 5 or greater relative to the wild-type strain with the empty vector, which was considered overexpression.

### Construction of the mutant and complemented strains

The mutant was constructed using homologous recombination with the pK18 vector plasmid. The upstream and downstream amplified gene fragments were ligated into the linear pK18 plasmid at the *Bam*HI and *Hind*III enzyme digestion sites. The recombinant plasmids were heat-shocked into *E. coli* DH5α competent cells. The cells were streaked on LB agar plates supplemented with 60 μg/mL gentamicin for single colony isolation and PCR verification. The successfully verified single colony was cultured for plasmid amplification, followed by plasmid extraction. Then, the plasmids were electroporated into PAO1 competent cells and screened on LB agar supplemented with 60 μg/mL Gm. Then, the resistant single colonies were patched on LB agar with 10% glucose. Potential mutants without gentamicin resistance were then screened and verified by RT‒qPCR. The complemented strain was constructed by electroporating the pAK1900-PA0982.1 recombinant plasmid into the mutant competent strain.

### Growth curve measurement

The growth curve measurement was performed by inoculating overnight cultures at a 1:100 dilution into 100 mL LB in a 96-well plate. The plate was then placed in a 37 °C incubator with shaking at 220 rpm. Samples were taken every 4 h to assess the OD_600_ from 0 h after inoculation.

### Biofilm formation assay

The biofilm formation assay was conducted according to a previously published method.^44^ A 2-μL aliquot of the overnight culture was inoculated into 2 mL of LB broth containing 150 μg/mL Cb and incubated at 37 °C for 16 h under static conditions. To harvest the biofilm, the planktonic layer was gently removed with a 1 mL pipette, and the tubes were washed 3 times with sterile distilled water, ensuring that the biofilm attached to the tube wall remained undisturbed. The biofilm was then stained with 2.5 mL of a 1% (w/v) crystal violet solution for 20 min. After staining, the crystal violet solution was discarded, and the tubes were rinsed with sterile distilled water to remove excess stain. Biofilm formation was quantified after air-drying the tube using 2 mL of 95% (v/v) ethanol to dissolve the crystal violet stain, and the optical density was measured at 590 nm (OD_590_). All analyses included 3 independent experiments.

### IntaRNA bioinformatic prediction and RNA electrophoretic mobility shift assay (EMSA)

In this study, we utilized IntaRNA (version 3.4.1) to predict potential interactions between small RNAs (sRNAs) and their mRNA targets. IntaRNA was installed via Conda on a Linux system. The specific command used for the prediction was “IntaRNA-q sRNA.fa -t rhli.fa”. We adhered to the default parameters set by IntaRNA: (--helixMinBP 2).

The EMSA technique was used to assess the binding affinity and specificity of the interactions under near-physiological conditions. The key steps of the EMSA protocol are as follows: RNA fragments were transcribed *in vitro* from DNA templates using a MAXIscript T7 Transcription Kit (Invitrogen, Thermo Scientific, USA). Increasing amounts of sRNA were incubated with *rhlI* 5′ UTR RNA at 37 °C for 20 min. Native polyacrylamide gel electrophoresis (PAGE) was then used to analyze the RNA–RNA interactions at 150 V for 90 min on ice.^45^

### Measurement of pyocyanin production

The strains were cultured overnight in LB broth with 150 μg/mL Cb at 37 °C with shaking at 220 rpm. Then, 20 μL cultures were inoculated into 2 mL fresh PYO medium, which consisted of 6 g/L alanine, 0.1 g/L K_2_HPO_4_, 2 g/L MgSO_4_, 1% glycerol, and 0.1 g/L C_6_H_5_FeO_7_, and sterile filtration through 0.22 μm membrane filters. After 16 h of incubation with shaking at 250 rpm, the bacterial pellet was harvested by centrifugation at 14,000 rpm for 15 min. The supernatant was transferred to a 96-well plate, and the OD_695_ of each sample was measured.

### Construction of translational reporter fusions and luciferase assay

To verify the direct regulation of *rhlI* by the sRNA PA0982.1, a translational fusion reporter was constructed according to previously published methods.^46^^,^^47^ The DNA fragment containing the *rhlI* promoter, the 5′ UTR, and the first 15 codons of the *rhlI* coding sequence was amplified and cloned and inserted into the *luxCDABE* reporter vector pKD-lux. The resulting recombinant plasmid *PrhlI*:*rhlI*_5’UTR+15aa_-*lux* was transformed into both the WT PAO1 and the *ΔPA0982.1* mutant strains. Luminescence production and OD_600_ were measured using a microplate reader (Thermo Scientific, USA). Relative luminescence units were normalized to cell density (luminescence/OD_600_).

### Quantification of N-butyryl-L-homoserine lactone (C4-HSL)

The concentration of C4-HSL in the bacterial culture supernatants was quantified using a commercial C4-HSL enzyme-linked immunosorbent assay Kit (MEIMIAN, MM-92778401, China) following the manufacturer’s instructions. Briefly, bacterial strains were cultured in LB medium overnight. The cultures were centrifuged at 12,000 rpm for 10 min, and the supernatants were collected and filtered through a 0.22 μm membrane to remove residual cells. Samples and standards were added to pre-coated microplate wells and incubated at 37 °C for 1 h. After washing with the wash solution 5 times, horseradish peroxidase (HRP)-conjugate and substrates were added for the colorimetric reaction. The absorbance was measured at 450 nm using a microplate reader. The C4-HSL concentration was calculated based on the standard curve followed by normalization relative to wild-type PAO1.

### Enrichment analysis

We clarified the distinct thresholds used for network construction versus general enrichment analysis. For the interactive analysis among sRNAs and target genes, we applied a stringent cutoff of adjusted *p* value (*P*_*adj*_ < 0.001, |log_2_FC| ≥ 3). For the sRNA–sRNA interaction network, a threshold of *P*_*adj*_ < 0.05 and |log_2_FC| ≥ 1 was utilized. Regarding the enrichment analysis, we employed clusterProfiler (version 4.17.0), a widely utilized R package designed for the interpretation of omics data. Differential expression analysis was performed using DESeq2, from which DEGs were identified. Subsequently, these DEGs were categorized into either upregulated or downregulated gene sets based on their fold changes. Each gene set was then analyzed using the ‘enricher’ function of clusterProfiler, employing a *p* value threshold of 0.05 for significance. The Gene Ontology (GO) annotation files necessary for this analysis were retrieved from the Pseudomonas Genome Database (https://www.pseudomonas.com/).

### Motility assay

The motility experiment was conducted according to the methodology outlined in a previous study.^48^ The overnight culture in LB broth was inoculated onto LB agar plates (8 g/L nutrient broth mix, 5 g/L glucose, and 0.5% agar) of 2.5 mL aliquots. Then, the cells were incubated statically for 16 h at 37 °C. Photographs of the plates were captured by the Bio-Rad ChemiDoc Touch Imaging System (Bio-Rad, CA, USA).

### Bacterial growth competition assay

With minor modifications to a previously published competition assay,^49^ overnight cultures of PAO1, sRNA overexpression strains (supplemented with 150 μg/mL Cb), and the *E. coli* strain with the pUC18 plasmid (supplemented with 12 mg/mL Gm) in LB were pelleted by gentle centrifugation at 5000 rpm for 3 min. Then, the pellets were resuspended in fresh LB broth by pipetting thoroughly and repeatedly washing 3 times. A 1:1 mixture of *P. aeruginosa* and *E. coli* was then prepared in a total volume of 1 mL LB broth. The mixture was then incubated at 37 °C with shaking at 220 rpm for 6 h. Subsequently, the culture was serially diluted and subjected to spot plating on LB agar supplemented with Gm to determine the colony-forming unit (CFU) ratios, assessing the growth competition.

### Statistical analysis

Statistical significance was determined using GraphPad Prism software. For comparisons between two groups, a two-tailed unpaired Student’s *t* test was performed. For comparisons involving three groups, a one-way analysis of variance (ANOVA) was conducted, followed by Dunnett’s multiple comparisons test. Data are presented as the means ± standard deviations (SD).^50^ Differences were considered statistically significant at ∗*p* < 0.05, ∗∗*p* < 0.01, and ∗∗∗*p* < 0.001. All experiments were independently replicated 3 times.

## Results

### Identification of 9 new intergenic RNAs in *P. aeruginosa* strain PAO1

To optimize sRNA detection in *P. aeruginosa*, we generated 4 RNA-seq libraries derived from ribosomal RNA–depleted RNA. To preserve native small RNAs and minimize recovery bias, total RNA was extracted using the Direct-zol method, which better retains short transcripts.^51^ The two biological replicates with two corresponding technical replicates were sequenced at different depths to assess the sensitivity of de novo sRNA detection (Fig. S1A). The two biological replicate libraries yielded average sequencing depths of 91.63 million and 24.68 million uniquely mapped reads to the *P. aeruginosa* genome, respectively. To identify potential sRNA candidates, we developed a computational pipeline to detect RNA signals in the intergenic region based on the mapping depth (Fig. S1B). Candidates were identified based on specific criteria: a minimum sRNA length of 50 bp, flanking anchor regions of at least 30 bp, and a minimum coverage fold-change of 1.5 relative to the anchors (code available at https://github.com/dengxinb2315/CCEP-sRNA/blob/main/code/sRNAprediction.Rmd). Overall, 1663 signal peaks were identified as intergenic RNA candidates across the 4 libraries. Subsequently, we performed a manual inspection of the BAM files using IGV to validate these candidates. To ensure reproducibility, selection was restricted to candidates exhibiting sharp, well-defined peak profiles distinct from background noise. As the transcriptional orientation of RNA cannot be retrieved directly from regular RNA sequencing (RNA-seq) data, we cross-referenced with published strand-specific LM-seq data.^52^ With this approach, the orientations of the known sRNAs RsmY and RsmZ were accurately identified (Fig. S2).

Specifically, 9 intergenic RNA loci that passed the above criteria were successfully validated by Northern blotting (Fig. 1A; Fig. S1C). For the intergenic region between PA4640 and PA4642, comparable signals were detected on both strands. However, only the reverse strand was detected by Northern blot. The sRNA RnpB was included as a positive control due to its stable expression and well-characterized size^34^ (Fig. 1A). These intergenic RNA candidates were further subjected to examination of their secondary structures, which were predicted with MXfold2 by calculating their minimum free energy (Fig. 1B). We observed that nearly all sRNAs featured stem–loop structures, which enhance stability and protect against nuclease degradation in the cell.^53^ In total, 9 candidate sRNAs were retained for further analyses (Fig. 1C).

**Figure 1:**
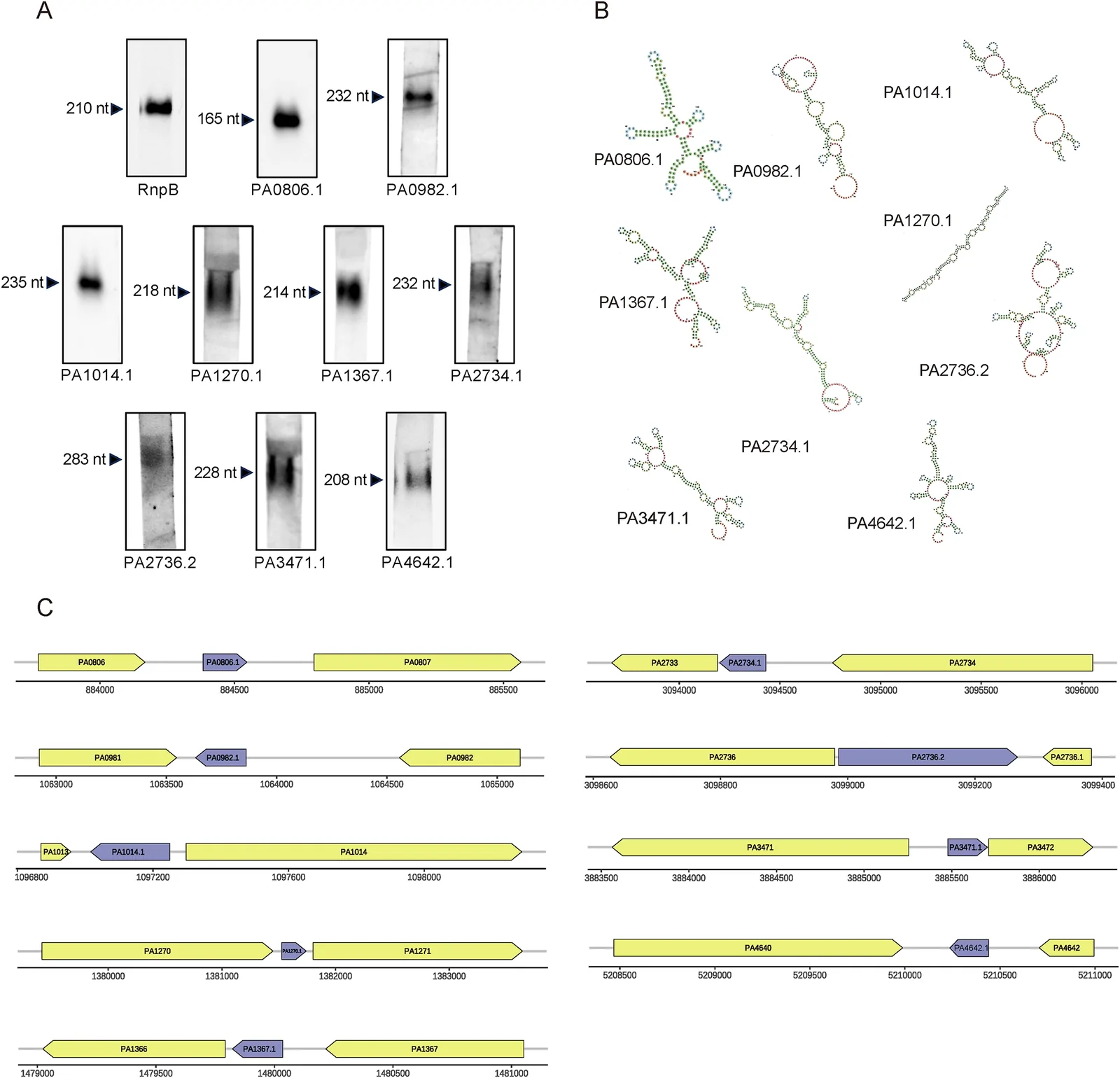
Prediction and validation of intergenic RNA signals in *P. aeruginosa*. **(A)** Validation of intergenic RNA expression through Northern blot analysis. The size of each sRNA is indicated on the left, with the arrowhead denoting the primary transcript observed in each blot. **(B)** Predicted secondary structures of the sRNAs generated using MXfold2 and visualized in a “structure” color scheme. **(C)** Genomic locations and orientations of identified sRNAs within non-coding regions of the *P. aeruginosa* PAO1 genome. The sRNAs are indicated in purple, and adjacent protein-coding genes are shown in yellow.

### Pan-sRNome involves 58 representative sRNAs across 484 *P. aeruginosa* strains

To understand all sRNAs in *P. aeruginosa*, we introduced the pan-sRNome (Fig. S3A). Specifically, the genome and RefSeq annotation files of 484 *P. aeruginosa* strains were extracted from NCBI. We collected sequences that were annotated with ‘ncRNA’ and ‘sRNA’, forming a pan-sRNome database that encompasses 1663 sequences. The length of the identified sRNAs in *P. aeruginosa* showed a multimodal distribution (Fig. S3B). These peaks suggest that the majority of sRNAs fall within the 100–400 nt range, which is consistent with typical bacterial sRNA lengths reported in previous studies.^5^ Utilizing MMseq, 58 representative sRNAs were highlighted (Table S1). We characterized the identity of 58 sRNAs in *P. aeruginosa* and identified the species-specific sRNAs that may play roles in their unique pathogenicity and ecological adaptations. Of the 58 sRNA homologs, 38 (65.52%) were identified as highly conserved in all 484 strains, with an alignment identity greater than 90%, and designated core sRNAs (Fig. 2A and B). Within this subset, 3 newly identified sRNAs (PA0806.1, PA3471.1, and PA4642.1) indicated their potential importance in cellular processes, as they were classified into core sRNAs. A total of 19 sRNA homologs (32.76%) were identified as accessory sRNAs (sRNAs found in two or more strains), including the other 6 newly identified sRNAs (PA0982.1, PA1014.1, PA1270.1, PA1367.1, PA2734.1, PA2736.2). Notably, none of the 9 newly identified sRNAs were identified as unique (target sRNA only found in 1 strain), indicating remarkable conservation (Fig. 2C).

**Figure 2:**
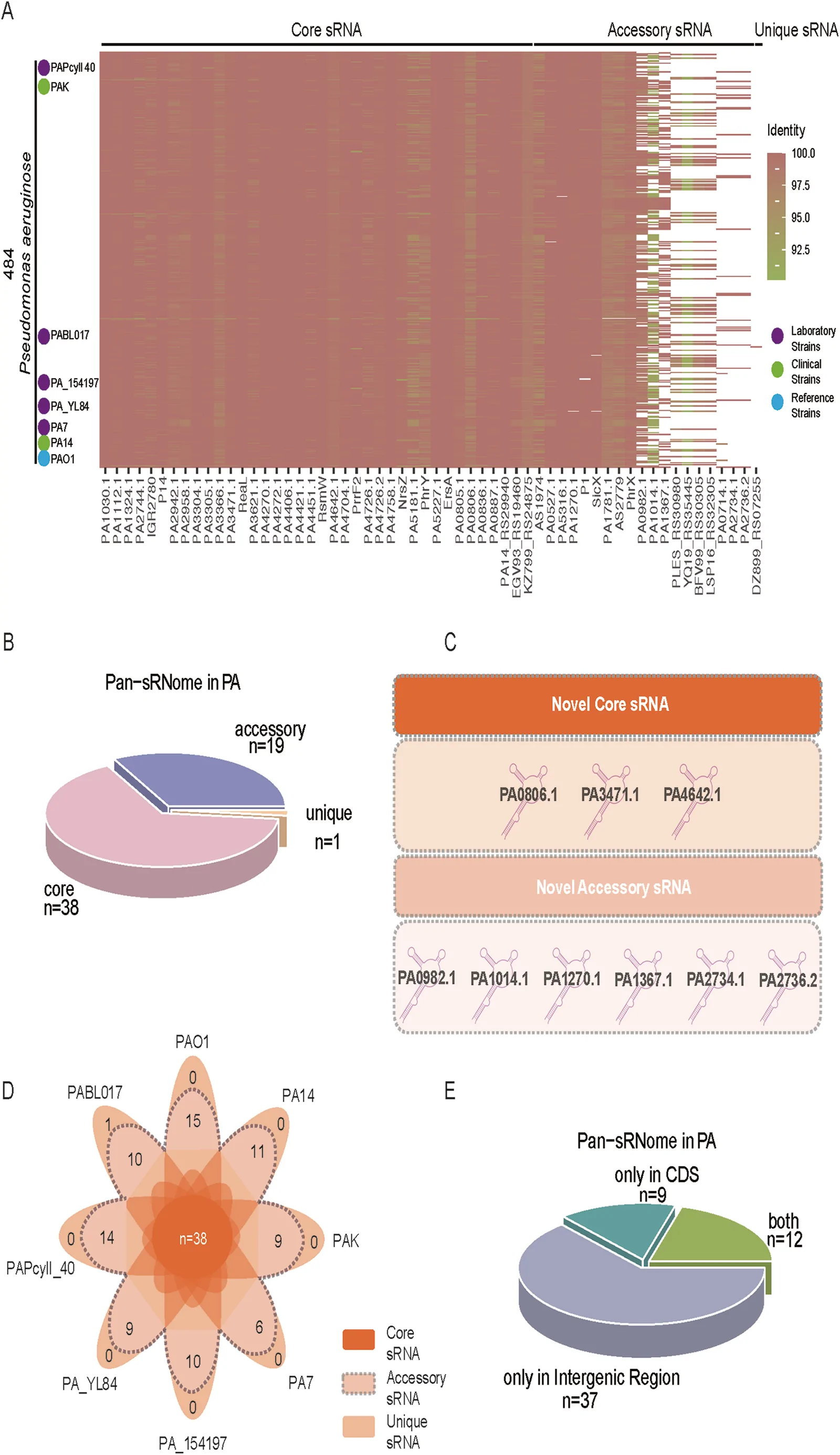
Overview of the pan-sRNome in *P. aeruginosa* strains. **(A)** Pan-sRNome profiling of *P. aeruginosa*. Classification of 58 annotated sRNAs from 484 *P. aeruginosa* genome data into 3 categories: core sRNAs (sRNAs found in all 484 strains), accessory sRNAs (sRNAs found in 2–483 strains), and unique sRNAs (sRNAs only found in 1 strain). Eight representative strains, including laboratory, clinical, and reference isolates, are highlighted, with nucleotide sequence identity indicated in the right-hand legend. **(B)** Distribution of the 58 annotated sRNAs within the *P. aeruginosa* Pan-sRNome, categorized by prevalence across strains. **(C)** Categorization of 9 new sRNAs within the Pan-sRNome of *P. aeruginosa*. **(D)** sRNA composition across 8 representative *P. aeruginosa* strains. A core set of 38 sRNAs was universally detected, while a unique sRNA was identified exclusively in the clinical strain PABL017. The number of accessory sRNAs varies from 6 to 15 across strains. **(E)** Genomic localization features of 58 sRNAs in *P. aeruginosa*. The pie chart shows the genomic positions of 58 representative sRNA sequence homologs, with 9 located within coding sequences (CDSs), 37 in intergenic regions (IGRs), and 12 spanning both CDSs and IGRs.

To investigate the prevalence of various sRNA categories among different *P. aeruginosa* strains from different sources, we selected 8 representative strains for analysis: 2 laboratory-adapted strains (PAK and PA14), 5 clinically derived strains (PAPcyll_40, PABL017, PA_154,197, PA_YL84, and PA7), and 1 reference strain (PAO1). Analysis of the sRNA distribution across 8 representative strains (Fig. 2D) revealed that while the core sRNome remained stable across all isolates, the accessory sRNome exhibited significant variability, ranging from 6 to 15 sRNAs per strain. Only 1 unique sRNA (DZ899_RS07255), a hammerhead ribozyme and one of several catalytic RNAs,^54^ was identified in the clinical strain PABL017. This distinct distribution pattern suggests that the variation in accessory sRNA content may underlie phenotypic diversity, including virulence, environmental adaptation, and antibiotic resistance in *P. aeruginosa*.^55^

Another interesting observation from our analysis is that the distribution of the sRNA homologs across the 484 *P. aeruginosa* strains was not exclusive to intergenic regions^30^; rather, some were mapped within coding DNA sequence (CDS) regions, and the proportions are presented as a pie chart in Figure 2E. Specifically, 9 sRNA homologs were mapped to CDS regions, and 12 sRNA homologs were variably aligned with either the IGRs or CDS regions across a subset of *P. aeruginosa* strains (Fig. S3C). These findings suggested that some of these sRNA sequences may encode functional peptides or proteins in other *P. aeruginosa* strains.

### Regulatory roles of 9 sRNAs in modulating virulence traits of *P. aeruginosa* PAO1

To evaluate the effects of the 9 newly identified sRNAs on the virulence traits of *P. aeruginosa*, sRNA overexpression was induced in strains, and differential expression analysis was performed to reveal the transcriptomic changes. Analysis of the RNA-seq data revealed that the overexpression of sRNA sequences resulted in variable modulation of gene expression profiles, leading to the upregulation or downregulation of downstream genes with |log_2_ FC| greater than 1 and *p* value < 0.05 (Fig. S4A). Subsequently, we constructed a comprehensive gene regulatory network comprising 1360 significant interactions among sRNAs and genes. Based on the regulatory network depicted in Figure 3A, the 9 newly identified sRNAs exerted diverse regulatory effects on virulence-related genes associated with 7 key phenotypic categories. In the “Motility & Biofilm” category, sRNAs such as PA2734.1, PA2736.2, and PA3471.1 downregulated the *pilE* and *pslL genes*, which are implicated in pilus assembly and biofilm formation. The “Pyocyanin” category showed that overexpression of sRNAs such as PA1014.1 and PA1367.1 was associated with the upregulation of *phz* genes, which are involved in pyocyanin (PYO) biosynthesis. These sRNAs positively regulated the genes involved in the “Motility & Biofilm” category; this coordinated upregulation likely contributes to the overall virulence and adaptability of the bacterium in infection. In the “T6SS” (Type VI Secretion System) category, overexpression of sRNAs such as PA0806.1, PA0982.1, PA1270.1, PA3471.1, and PA4642.1 resulted in reduced transcript levels of *hcpA*, *hcpB*, and *hcpC* genes, which are essential for interbacterial competition and host interaction. Except for PA2734.1, PA2736.2, and PA4642.1, the remaining 6 sRNAs were involved in the regulation of Type III secretion system (T3SS)-related genes, such as *exsE*, *popN*, *exoS*, *exoY*, *pscG*, *pscD*, *pscJ*, *pscV*, *exsC*, and *pscT*, which are critical for virulence factor secretion. Last, for the “Quorum Sensing” category, overexpression of sRNA PA3471.1 coincided with altered expression levels of *rhlA*, *lasB*, *rhlC*, *pqsE*, *pprB*, and *mpaR*, influencing quorum sensing and associated virulence pathways.

**Figure 3:**
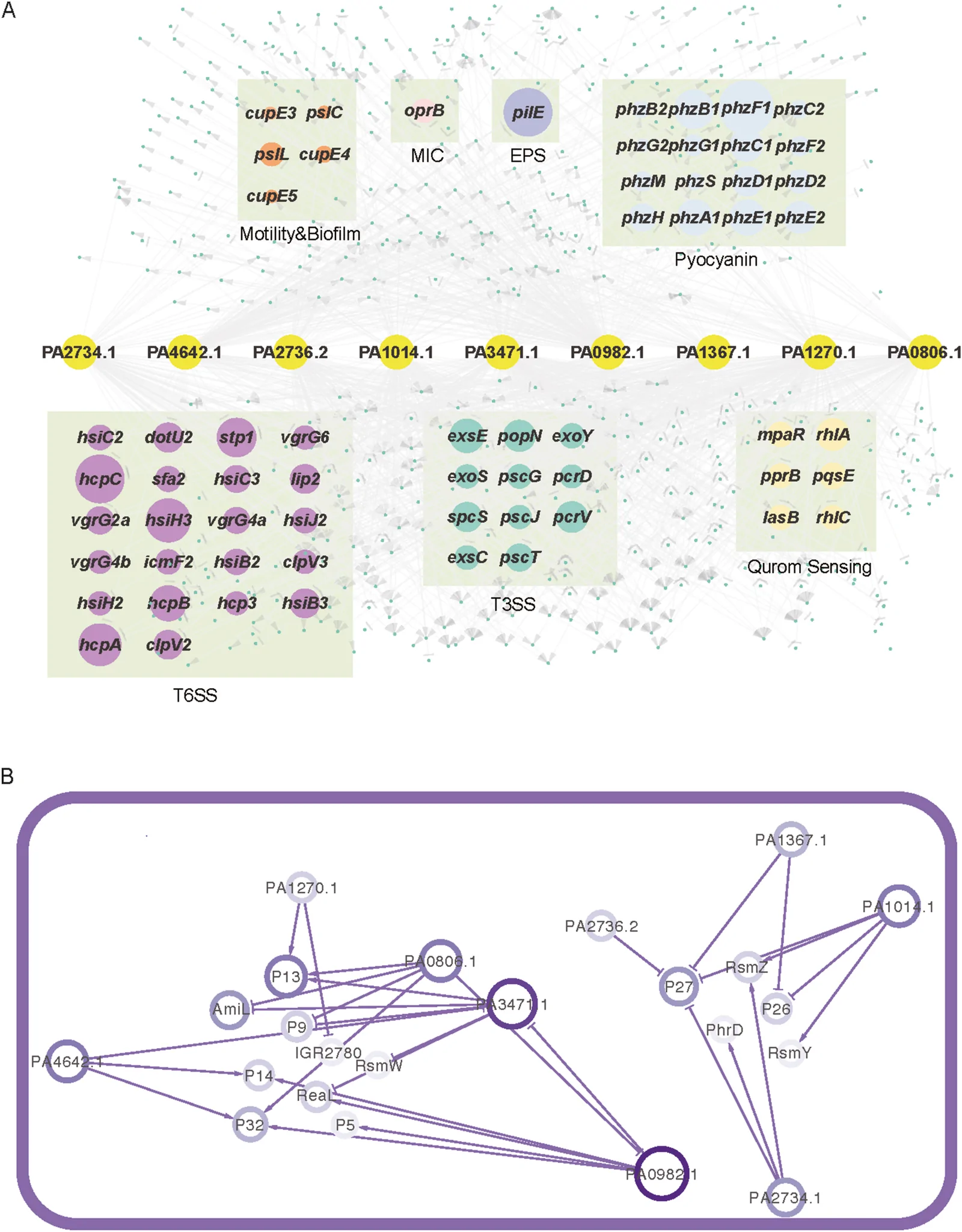
Interactive analysis among sRNAs and target genes. **(A)** The sRNA regulatory network highlights 7 virulence-associated functional gene clusters, each enclosed in a distinct box. Solid arrows indicate upregulation, while T-shaped arrows denote downregulation. (cutoff: adjusted *p* value [*P*_*adj*_] < 0.001, |log_2_ FC| ≥ 3). **(B)** sRNA–sRNA interaction network highlighting interactions among 9 sRNAs and known sRNAs in *P. aeruginosa* (cutoff: *P*_*adj*_ < 0.05, |log_2_FC| ≥ 1). Node color intensity reflects the number of regulatory connections, with darker shades signifying a higher degree of interaction.

### Regulatory interactions among sRNAs in *P. aeruginosa*

To explore the broader regulatory landscape of the 9 novel sRNAs in *P. aeruginosa*, we examined potential sRNA–sRNA regulations that may expand regulatory relationships. Small RNAs can form complex transcriptional networks by modulating each other’s expression, amplifying regulatory effects. Using RNA-seq data, we mapped the interactions among 9 new sRNAs and other experimentally verified functional sRNAs, revealing a network of a total of 23 validated sRNAs in *P. aeruginosa* (Fig. 3B). Notably, most newly identified sRNAs exhibited interactions with multiple other sRNAs, revealing a multilayered regulatory network. Within this sRNA–sRNA network, PA1014.1 emerged as a potential node in the regulatory web. RNA-seq data indicated that its overexpression coincided with increased levels of RsmY and RsmZ, which are known to enhance quorum sensing (QS) gene expression through the GacS/GacA two-component system.^16^ Conversely, transcriptomic analysis suggested repression of P27 compared to WTEV, an sRNA known to inhibit the Rhl QS system.^56^ To validate these observations, we performed RT‒qPCR, which confirmed that overexpression of PA1014.1 significantly reduced P27 levels. Similarly, PA2734.1 overexpression was associated with upregulated RsmZ and repressed P27 levels in RNA-seq data, a trend that was successfully validated by RT‒qPCR (Fig. S4B). Collectively, these findings highlight the complexity of the QS regulatory landscape, suggesting that these sRNAs modulate gene expression through multilayered sRNA–sRNA and sRNA-mRNA interplay in *P. aeruginosa*.

### sRNA PA0982.1 modulates the QS system by binding to the *rhlI* 5′ UTR and altering *rhlR* expression

The *rhl* QS system in *P. aeruginosa* is a critical component of the bacterial communication network, which regulates the expression of genes for virulence and biofilm formation.^57^ The published RNA interaction by ligation and sequencing data reveal a direct RNA–RNA interaction between the intergenic region of the *rhlI-rhlR* gene and the intergenic region of the *PA0981-PA0982 gene*.^36^ This experimental evidence aligns with our prediction from the IntaRNA tool, which indicated that the sRNA PA0982.1 directly binds to the 5′ UTR of the *rhlI* gene, with a binding free energy of −12.97 kcal/mol, suggesting a stable interaction (Fig. 4A). This interaction was further confirmed by RNA EMSA, which demonstrated the direct binding of the sRNA PA0982.1 to the 5′ UTR of *rhlI in vitro* (Fig. 4B). Additionally, RNA EMSA revealed the interaction between the sRNA PA0982.1 and the RNA chaperone Hfq, indicating a potential role of Hfq in stabilizing the sRNA PA0982.1 or facilitating its interaction with the *rhlI* mRNA (Fig. 4C).

**Figure 4:**
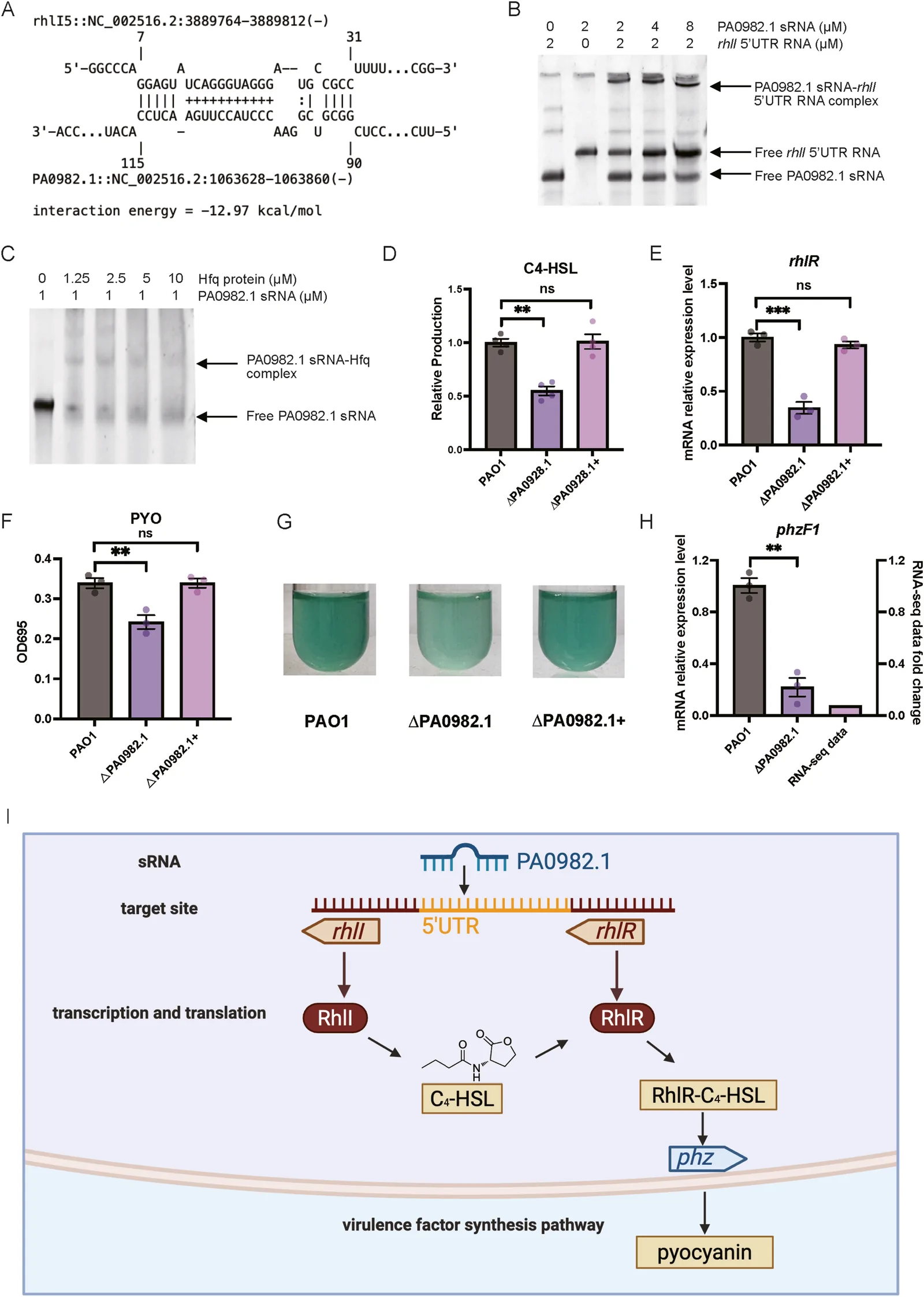
Molecular and functional validation of PA0982.1 regulation of the *rhl* QS system in *P. aeruginosa*. **(A)** Predicted RNA–RNA interaction site between PA0982.1 and the *rhlI* 5′ UTR. **(B)** RNA EMSA of PA0982.1 binding to the *rhlI* 5′ UTR *in vitro*, with increasing PA0982.1 concentrations (0, 2, 4, 8 μM) shifting the complex formation, alongside free *rhlI* 5′ UTR and PA0982.1 sRNA. **(C)** Protein‒RNA EMSA interaction between PA0982.1 and Hfq, with increasing PA0982.1 concentrations forming a PA0982.1-Hfq complex, alongside free PA0982.1 RNA. **(D)** Quantification of C4-HSL production levels in the sRNA PA0982.1 mutant (Δ*PA0982.1*) and complemented strains (Δ*PA0982.1*+), measured relative to wild-type (WT) PAO1. **(E)** Relative mRNA expression levels of *rhlR* in WT, Δ*PA0982.1*, and complemented strains, determined by RT‒qPCR. **(F)** PYO production in WT, Δ*PA0982.1*, and complemented strains measured at OD_695_. **(G)** Visual representation of PYO production in culture media of WT, Δ*PA0982.1*, and Δ*PA0982.1*+. **(H)** Relative mRNA expression levels of *phzF1* in WT, Δ*PA0982.1*, and complemented strains, determined by RNA-seq and validated by RT‒qPCR. **(I)** Working mechanism of sRNA PA0982.1 in regulating the *rhl* QS system and pyocyanin production.

To specifically investigate the regulatory mechanism of sRNA PA0982.1 on the *rhl* QS system, we knocked out sRNA PA0982.1 (Δ*PA0982.1*) to assess its direct role under native conditions. Functional analysis by enzyme-linked immunosorbent assay showed that disruption of sRNA PA0982.1 significantly reduced the production of C4-HSL, a key quorum-sensing molecule synthesized by RhlI, compared to WT PAO1 and the complemented strain (Fig. 4D). To determine whether this reduction was due to transcript degradation, we measured *rhlI* mRNA levels by RT‒qPCR but found no significant difference between the WT and ΔPA0982.1 strains (Fig. S4C). To further confirm the direct regulation of *rhlI* by PA0982.1, we constructed a translational reporter fusion containing the *rhlI* promoter, 5′ UTR, and the first 15 codons fused to the *lux* operon. This reporter was introduced into both the WT and *ΔPA0982.1* strains. As shown in Figure S4D, the luminescence activity in the *ΔPA0982.1* mutant was significantly reduced compared to that in the WT strain. This result provides evidence that the sRNA PA0982.1 positively regulates *rhlI* expression, likely by stabilizing the transcript or facilitating translation initiation. Correspondingly, the mRNA level of *rhlR*, the downstream QS regulator, was markedly decreased in Δ*PA0982.1* (Fig. 4E). These findings suggested that the sRNA PA0982.1 positively regulates the *rhl* QS pathway.

Phenotypic analysis further revealed that Δ*PA0982.1* exhibited significantly lower PYO production than PAO1 and the complemented strain (Fig. 4F and G). The DEGs from RNA-seq and RT‒qPCR data confirmed that the expression of *phzF1*, a key gene involved in PYO biosynthesis, was downregulated in Δ*PA0982.1* (Fig. 4H). Taken together, these results demonstrated that sRNA PA0982.1 regulates the translation of *rhlI* through interaction with its 5′ UTR, thereby modulating C4-HSL synthesis and downstream QS regulatory networks, ultimately influencing PYO production (Fig. 4I).

### sRNAs PA1014.1, PA2734.1, and PA2736.2 promote PYO production *in vitro*

To determine whether the observed transcriptional changes were attributable to general effects on bacterial fitness, we first performed growth curve analyses for all sRNA overexpression strains. Overexpression did not alter growth rates under the tested conditions, indicating that the transcriptomic effects are unlikely to result from nonspecific growth defects (Fig. S5A). GO analysis revealed that overexpression of PA1014.1, PA2734.1, and PA2736.2 upregulated phenazine biosynthesis (Fig. 5A–C). Transcriptomic analysis indicated that the expression of key phenazine biosynthesis genes, including *phzB2*, *phzG2*, and *phzS*, was significantly enhanced in these sRNA overexpression strains. To verify this, we measured relative mRNA expression levels by RT‒qPCR and confirmed the upregulation of *phzB2*, *phzG2*, and *phzS* genes, consistent with the RNA-seq data (Fig. 5D, G, J). Consequently, we hypothesized that this transcriptional upregulation would lead to increased PYO production. To test this hypothesis, we measured PYO production via a colorimetric analysis of the bacterial cultures. The PYO production of strains overexpressing PA1014.1, PA2734.1, and PA2736.2 was noticeably darker than that of the control (Fig. 5E, H, K), with *p* values of 0.0036, 0.0011, and 0.0055, respectively (Fig. 5F, I, L). These findings indicated that overexpression of sRNAs PA1014.1, PA2734.1, and PA2736.2 is associated with increased PYO production, concomitant with upregulation of phenazine biosynthesis genes.

**Figure 5:**
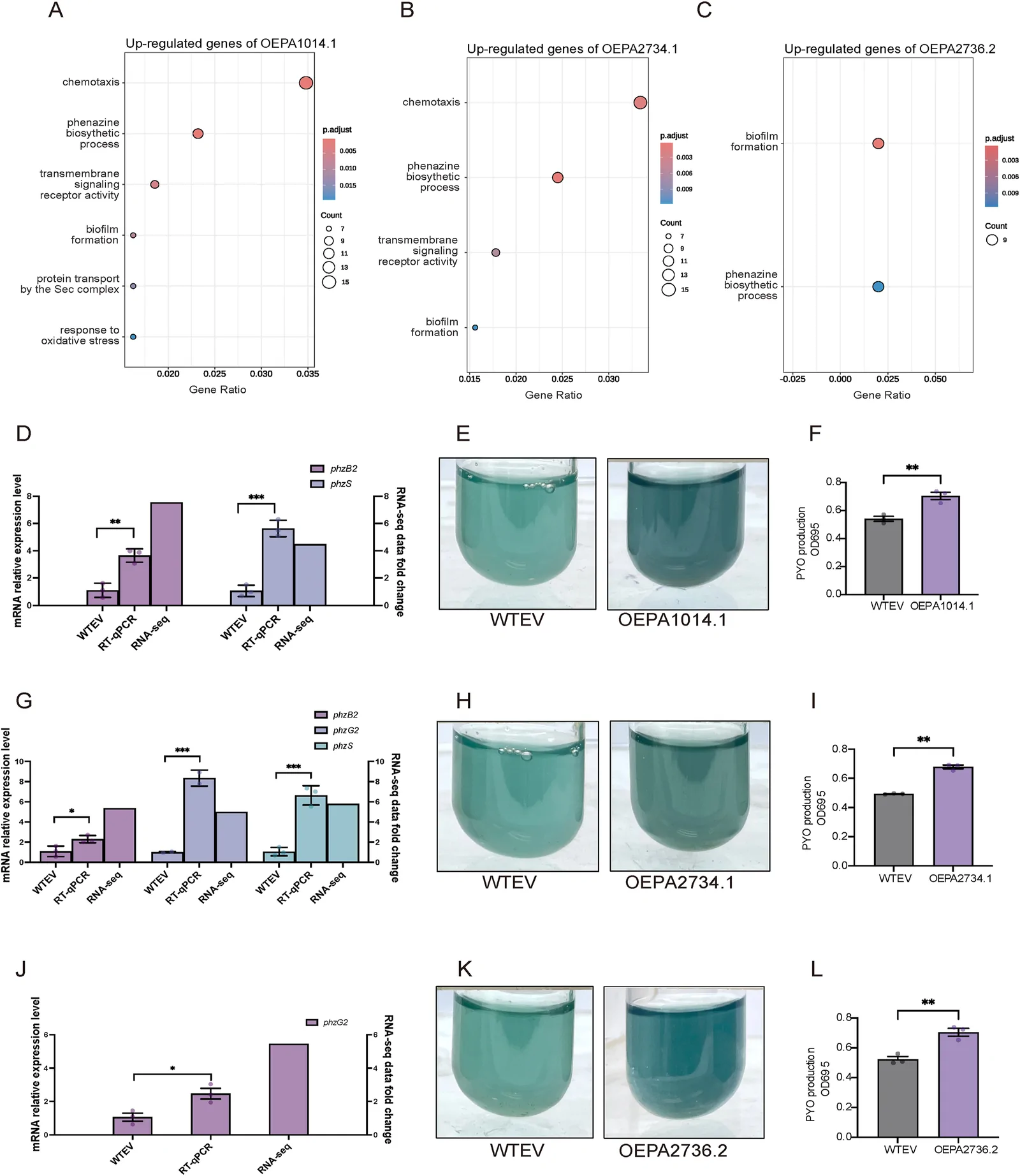
Functional regulation of PYO Production by sRNAs PA1014.1, PA2734.1, and PA2736.2. **(A–C)** Bubble plot depicting enriched GO terms for upregulated DEGs in PA1014.1, PA2734.1, and PA2736.2 overexpression (OE) strains compared to WTEV. **(D)** Detection of *phzB2* and *phzS* gene expression in the WTEV and PA1014.1 overexpression strains by RT‒qPCR. **(E, F)** PYO production is positively regulated by PA1014.1, as evidenced by colorimetric analysis comparing WTEV and PA1014.1 overexpression strains. **(G)** Detection of *cupB2*, *phzG2*, and *phzS* gene expression in the WTEV and PA2734.1 overexpression strains by RT‒qPCR. **(H, I)** PA2734.1 positively regulated pyocyanin production. **(J)** Detection of *phzG2* gene expression in the WTEV and PA2736.2 overexpression strains by RT‒qPCR. **(K, L)** PA2736.2 positively regulated pyocyanin production.

### Impact of sRNAs PA1014.1, PA2734.1, and PA2736.2 on biofilm formation and motility

PYO production is known to promote biofilm formation through QS-dependent pathways in *P. aeruginosa*.^58^ Given the complex interplay between PYO production and biofilm formation, we investigated how these sRNAs modulate biofilm formation to further elucidate their contributions to *P. aeruginosa* virulence. Biofilm assays revealed that strains overexpressing PA1014.1 and PA2734.1 exhibited significantly enhanced biofilm formation compared to WTEV (*p* < 0.001) (Fig. 6A, B, D, E). RNA-seq analysis of DEGs identified upregulated biofilm-associated genes in sRNA overexpression strains. Overexpression of PA1014.1 resulted in increased levels of *cupE5*, a crucial component of biofilm matrix formation.^59^ Similarly, PA2734.1 overexpression increased *cupE3* and *cupE4* expression by fourfold and eightfold, respectively, and upregulated *pslC*, which encodes a polysaccharide essential for biofilm structure.^60^ RT‒qPCR validated these expression changes, consistent with the RNA-seq data (Fig. 6C, F). These findings indicated that overexpression of sRNAs PA1014.1 and PA2734.1 is associated with enhanced biofilm formation, accompanied by upregulation of *cupE* and *pslC* genes, consistent with the transcriptomic data. These results supported a potential role of sRNAs in QS-related virulence pathways.

**Figure 6:**
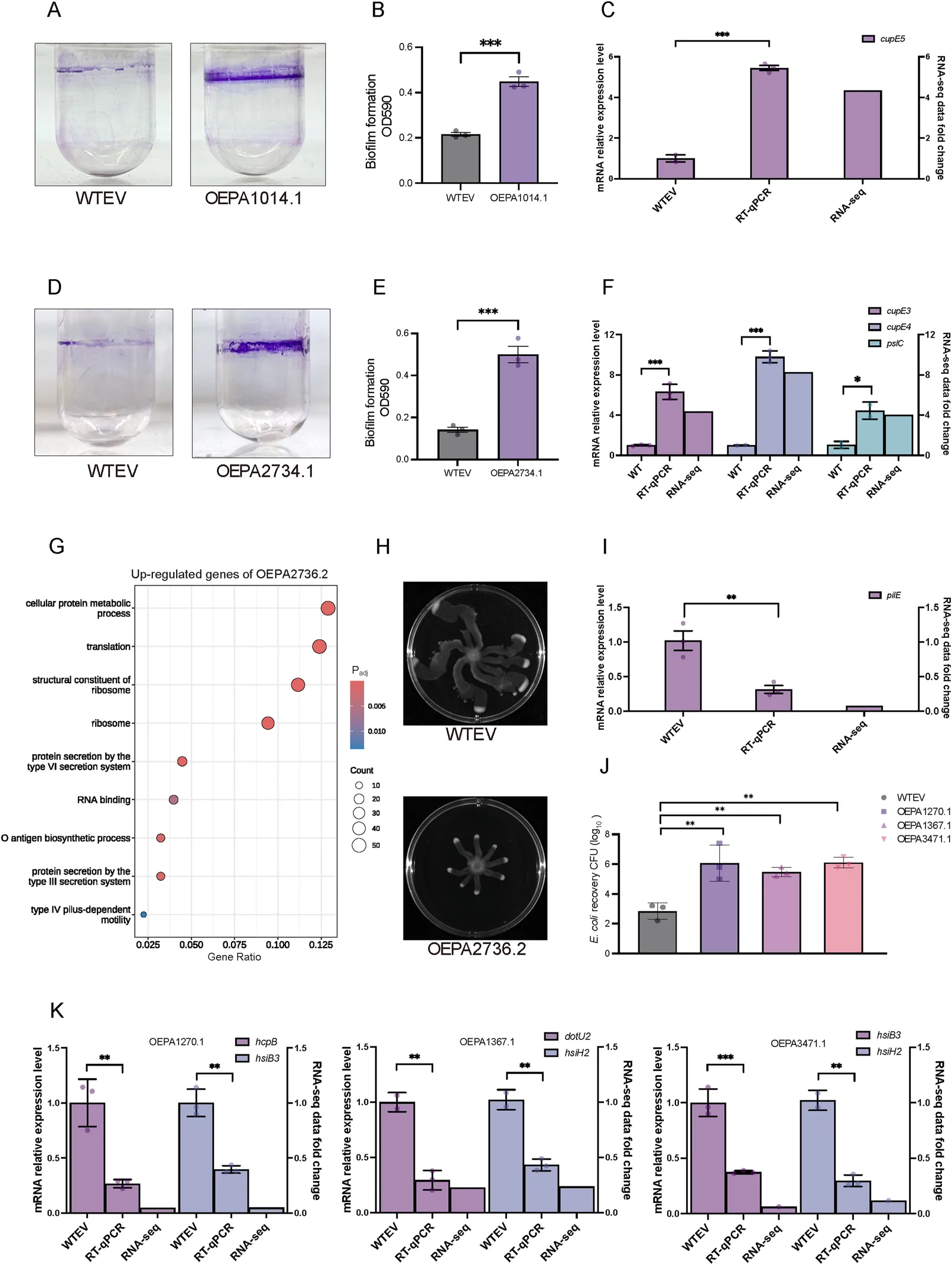
Phenotypic screening of virulence-related traits in sRNA overexpression strains. **(A, B)** Biofilm formation assays comparing WTEV and the PA1014.1 overexpression strain. **(C)** Detection of the *cupE5* gene expression in the WTEV and PA1014.1 overexpression strains by RT‒qPCR. **(D, E)** Biofilm formation assays comparing the WTEV and PA2734.1 overexpression strains. **(F)** Detection of *cupE3*, *cupE4*, and *pslC* gene expression in the WTEV and PA1014.1 overexpression strains by RT‒qPCR. **(G)** GO terms for downregulated DEGs comparing PA2736.2 overexpression strains to WTEV. **(H)** Swarming motility detection in the PA2736.2 overexpression strain compared to WTEV. **(I)** Detection of gene *pilE* expression in the WTEV and PA2736.2 overexpression strains by RT‒qPCR. **(J)** Bacterial growth competition assay between sRNA overexpression strains and *E. coli*. **(K)** Detection of T6SS-related gene expression in WTEV and PA1270.1, PA1367.1, and PA3471.1 overexpression strains by RT‒qPCR.

As motility represents a key virulence determinant essential for bacterial invasion,^61^ we next investigated the roles of sRNAs in regulating swarming motility, another critical QS-mediated virulence trait in *P. aeruginosa*. Swarming motility, which is essential for bacterial invasion and colonization, often exhibits an inverse relationship with biofilm formation, reflecting a dynamic balance in virulence strategies.^62^^,^^63^ To elucidate how sRNAs modulate motility, we analyzed their impact on motility-related gene expression and phenotypic outcomes. GO analysis of the PA2736.2 overexpression strain indicated that type IV pilus-dependent motility-related genes were enriched and downregulated (Fig. 6G). The swarming motility of the PA2736.2 overexpression strain was reduced compared to that of WTEV (Fig. 6H). RNA-seq identified a significant reduction in *pilE* expression, encoding a type IV fimbrial biogenesis protein, which was validated by RT‒qPCR (Fig. 6I). These results suggested that overexpression of sRNA PA2736.2 correlates with reduced swarming motility and downregulation of *pilE*, consistent with the observed transcriptomic patterns. Similarly, RNA-seq showed a greater than threefold reduction in *pilE* expression in the PA2734.1 overexpression strain, despite no GO enrichment for motility genes. This observation was experimentally confirmed by motility assays on swarming agar and supported by the reduction in *pilE* mRNA expression level, as depicted in Figure S5B and S5C.

To further characterize the regulatory impacts of sRNAs PA1014.1, PA2734.1, and PA2736.2, we performed Cluster of Orthologous Groups (COG) analysis on proteins from their overexpressing strains (Fig. S6). This analysis aimed to uncover how their regulatory effects extend to metabolic and adaptive processes supporting virulence. Beyond proteins in unassigned (S) and general function prediction (R) categories, significant enrichment was observed in energy production and conversion (C) and amino acid transport and metabolism (E) categories for upregulation (*p* < 0.05). This upregulation suggests heightened energy demands and metabolic adaptations, supporting the enhanced virulence traits observed. These findings underscore that sRNAs PA1014.1, PA2734.1, and PA2736.2 are involved in a multifaceted regulatory network, integrating virulence factor expression with metabolic optimization to enhance pathogenicity. In summary, our findings collectively suggested that sRNAs PA1014.1, PA2734.1, and PA2736.2 are associated with alterations in biofilm formation and motility, pointing to potential roles in the regulation of *P. aeruginosa* virulence.

### Overexpression of sRNAs PA1270.1, PA1367.1, and PA3471.1 suppresses T6SS components and reduces competitive fitness

The T6SS, a key virulence factor in *P. aeruginosa* acute and chronic infections, mediates bacterial competition and host interactions. Upon observing the downregulation of T6SS components in sRNA overexpression strains, we investigated how sRNAs PA1270.1, PA1367.1, and PA3471.1 modulate this system and impact interbacterial fitness (Fig. S5D–S5F). To assess the functional impact on interbacterial competition, we conducted a growth competition assay comparing the sRNA overexpression strains PA1270.1, PA1367.1, and PA3471.1 with *E. coli*. As shown in Figure 6J, *E. coli* exhibited a competitive advantage, with higher colony-forming unit (CFU) ratios compared to the control WTEV, indicating reduced competitive ability in sRNA-overexpressing strains. To identify the underlying mechanisms, we analyzed the expression of T6SS-related genes, including *hsiB3* (encoding Hcp secretion island-III T6SS), *hcpB*, *hsiH2* (conserved genes related to the key structure of H2-T6SS), and *dotU2* (encoding a T6SS functional protein). RT‒qPCR confirmed significant downregulation of *hcpB* and *hsiB3* in PA1270.1, *dotU2* and *hsiH2* in PA1367.1, and *hsiB3* and *hsiH2* in PA3471.1 overexpression strains compared to WTEV (Fig. 6K). These results demonstrate that the overexpression of these sRNAs leads to the downregulation of T6SS components. Given the established role of the T6SS in interbacterial competition, our data suggest that this downregulation likely contributes to the observed reduction in competitive fitness in *P. aeruginosa*, consistent with a strategy of diversifying virulence traits.

### sRNA homologs exhibit conserved distribution and selective evolutionary patterns across bacteria and archaea

Understanding the phylogenetic distribution and evolutionary history of sRNA homologs is crucial for elucidating their functional roles across bacterial species.^1^ Accordingly, we performed a phylogenetic comparison of 9 sRNAs across 23 species, including gram-positive bacteria such as *Streptococcus pneumoniae*, *Listeria monocytogenes*, and *Bacillus cereus*; gram-negative bacteria such as *Chlamydia trachomatis*, *Klebsiella pneumoniae*, and *Salmonella enterica*; and several archaeal species (Fig. 7). In terms of distribution, the PA4642.1 homolog was the most abundant, detected in 14 out of 23 species of bacteria and archaea, reflecting its broad conservation and suggesting a conserved functional role. Due to the close evolutionary relationship between *P. syringae* and *P. aeruginosa*, all sRNA homologs were found in the *Pseudomonas syringae* pv. *tomato* DC3000 or *Pseudomonas savastanoi* pv. *phaseolicola* 1448A strain, reflecting their close phylogenetic proximity. To further assess conservation within *P. aeruginosa*, we analyzed sRNA homologs across 200 *P. aeruginosa* strains. Sequence alignments revealed identity scores of 80%–100% and alignment scores of 100–500, indicating robust intraspecies conservation. The majority of homologs were located within CDS regions, reinforcing their potential integration into coding genes or regulation of adjacent sequences, potentially influencing translation or mRNA stability^64^ (Fig. S7). This high conservation within *P. aeruginosa* supported the hypothesis of shared sRNA-mediated regulatory mechanisms observed across the *Pseudomonas* genus. The long branch distances in the phylogenetic tree indicated weak phylogenetic relationships among the bacteria. Notably, homolog presence decreased with phylogenetic distance, except between *Nocardia cyriacigeorgica* and *Neisseria meningitidis*. This exception suggested selective conservation of sRNA functions despite weak evolutionary relationships, possibly due to shared ecological niches or horizontal gene transfer.

**Figure 7:**
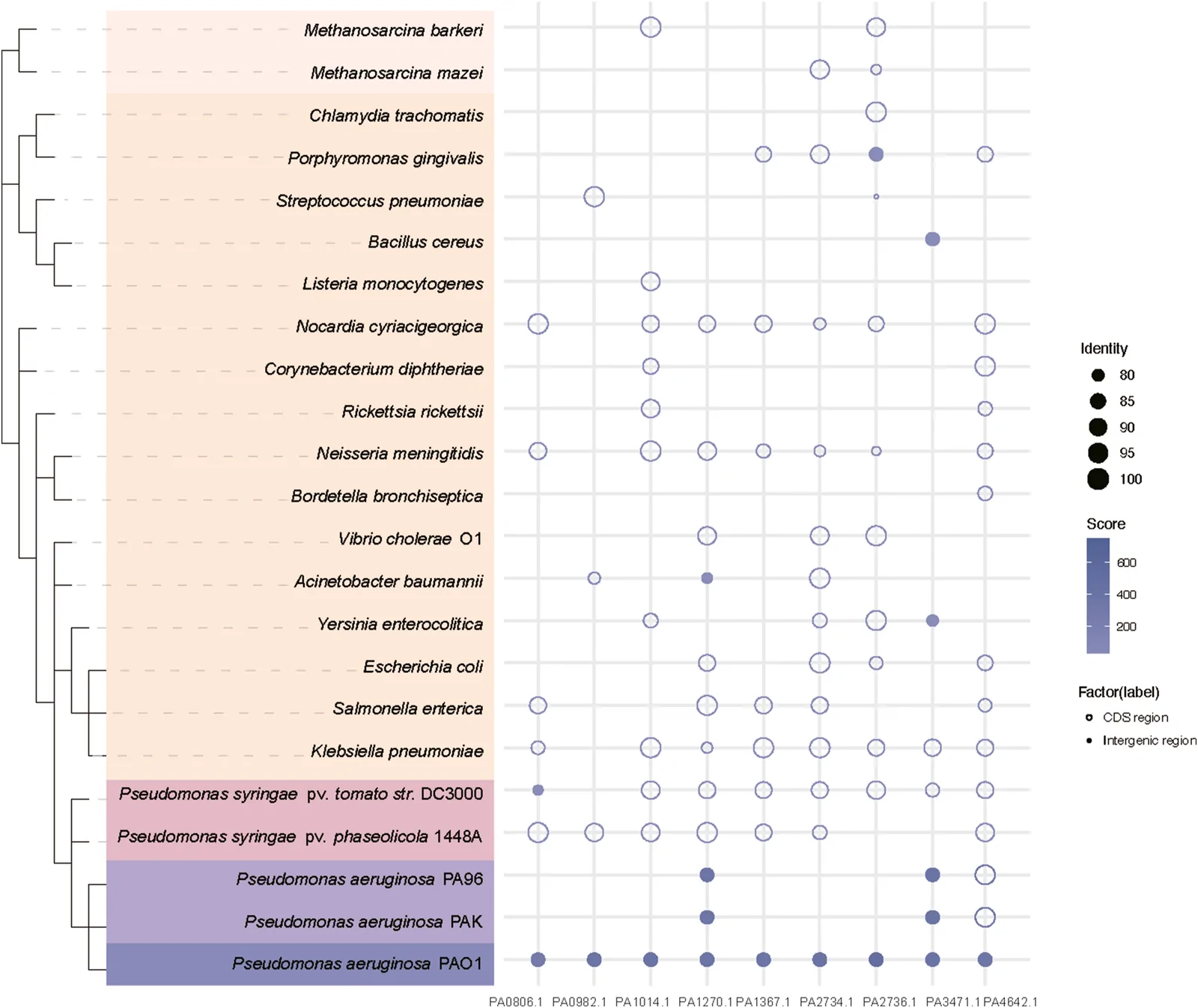
Phylogenetic analysis and dot plot visualization of sRNAs across *Pseudomonas* and different bacterial species. The phylogenetic tree was generated from the NCBI Taxonomy Browser and visualized by iTOL v6. The scale bar indicates genetic distance, and the branches show the phylogenetic relationships among the *Pseudomonas* species, other bacterial species, and archaea. The identity plot illustrates the conservation of each sRNA sequence. The horizontal and vertical axes represent the 9 sRNA sequences and 23 representative bacterial species, respectively. Dots indicate the presence of sRNA sequences in the IGRs, and hollow circles represent the location of sRNAs within a CDS region of the bacterial genome. The size of each dot and hollow circle correlates with the sequence identity, which refers to the extent to which nucleotides are identical at specific positions. The present identity represents a weighted measure of sRNA sequence similarity in sequence alignment analyses.

## Discussion

To date, genome-wide sRNA searches have been conducted in only a few bacterial species, and most of them are concentrated in *E. coli*.^65^ However, only approximately 40 sRNAs have been annotated in PAO1. In this study, we developed a new approach, “CCEP”, and identified the sequences and functions of 9 novel intergenic sRNAs. To better understand how sRNAs exert post-transcriptional regulation of gene expression, we developed regulatory networks to highlight the roles of newly identified sRNAs. Interestingly, we showed that the sRNA PA0982.1 modulates the QS system by directly binding to the *rhlI* 5′ UTR, and 3 sRNAs (PA1014.1, PA2734.1, and PA2736.2) were potential modulators of QS, as evidenced by enhanced PYO production, biofilm formation, and restricted motility. We further observed that the influence of these sRNAs on the QS system was accompanied by changes in the expression of *phz, cup, psl,* and *pil*-related genes. The overexpression of PA1270.1, PA1367.1, and PA3471.1 led to the suppression of T6SS components, which was associated with reduced competitive fitness (Table S2). Further research is necessary to confirm the virulence-suppressing properties associated with the identified targets of sRNAs in *P. aeruginosa* and to gain a more comprehensive understanding of the mechanisms of action.

Previous studies have already shown the relationship between sRNAs and the QS system. The overexpression of the sRNA PhrD in PAO1 revealed an increase in pyocyanin production, which is attributed to the positive regulation of the quorum sensing regulator RhlR.^66^ Similarly, the overexpression of sRNA PhrS also resulted in increased production of PYO.^67^ A deletion mutant of the sRNA AmiL led to an increase in PYO production, while AmiL was thought to be inhibited by RhlR.^68^ Taken together, the above observations reflect the indirect regulatory effects of sRNAs on virulence. In this study, we investigated the direct interactions between PA0982.1 and the QS-related gene *rhlI*. We also explored the roles of sRNAs in PAO1 and their potential interactions independent of Hfq, a known RNA-binding protein that typically facilitates sRNA–mRNA interactions.^69^

In the past, the significant challenge in sRNA identification was the variability in basal expression levels under different growth conditions, which limits the detection of annotated sRNAs. For example, in the human pathogen *Neisseria gonorrhoeae*, the amounts of identifiable sRNAs vary between iron-replete and iron-depleted growth conditions.^19^^,^^70^^,^^71^ Our sRNA predictions in *P. aeruginosa* were made only under the nutrient-rich environment in LB, which may lead to a variation in the number of sRNAs identified in our study compared with the numbers predicted under different culture conditions.

Further investigation into the sRNA–sRNA interaction network is essential, particularly regarding the roles of Hfq and other potential chaperone proteins. Our findings suggest that alternative regulatory mechanisms may govern these interactions, potentially uncovering new dimensions of post-transcriptional regulation. This view is supported by studies such as that of Holmqvist et al, which identified ProQ proteins as mediators of sRNA interactions, thus expanding our understanding of RNA-based regulatory networks in bacteria.^72^ Together, these insights deepen our knowledge of bacterial adaptability and pathogenicity.

Our investigation of the sRNome provided insights into conserved regulatory mechanisms in *P. aeruginosa*. As the taxonomical investigation of the sRNome has not yet been achieved, this work is the first to characterize a pan-sRNome containing completely verified sRNAs, thus filling a gap in the field. In the *P. aeruginosa* pan-genome, the core gene sequences involved in central and conserved processes, such as metabolism and pathogenicity, are highly conserved.^73^ However, these core genes typically constitute only a small fraction of the entire pan-genome (1%–15% of the total).^74^^,^^75^ These genes are generally not considered to be viable targets for further clinically related investigations. In contrast, in our pan-sRNome analysis, the core sRNAs were found to constitute 65.52% of the total sRNAs. This observation reveals that the core sRNAs in the pan-sRNomes were more highly conserved than the core genes in the pangenomes of *P. aeruginosa* species. Interestingly, the 3 sRNAs with verified regulatory functions were identified within the accessory sRNA group, underscoring their contributions to the diversity of pathogenic traits observed across different strains. We therefore hypothesize that during evolutionary development, the loss of these functional sRNAs may have facilitated the transition from low virulence to high virulence, as exemplified by comparing PAO1 with clinical strains.

However, it is important to acknowledge the limitations of extrapolating functional findings from the laboratory strain PAO1 to the broader *P. aeruginosa* population. While PAO1 serves as a model, our pan-sRNome analysis highlights a clear distinction between conserved and strain-specific regulatory elements. Core sRNAs, such as PA3471.1, are present in the majority of strains, suggesting that their regulatory roles likely represent conserved functions essential for general bacterial physiology. In contrast, accessory sRNAs such as PA1014.1 and PA1270.1 may drive strain-specific adaptations, allowing specific lineages to fine-tune virulence traits such as biofilm formation or interbacterial competition in response to unique environmental niches. Furthermore, even among conserved sRNAs, variations in basal expression levels or sequence divergence in target mRNA UTRs could lead to differential regulatory outcomes in clinical isolates. Therefore, while this study establishes a functional framework in PAO1, future validation in diverse clinical isolates is necessary to fully define the prevalence of these sRNA-mediated networks.

In summary, this study represents a significant advancement in our understanding of the roles of sRNAs in the regulatory networks of *P. aeruginosa* strain PAO1. Our CCEP approach successfully predicted and identified new sRNAs and expanded the pan-sRNome of this pathogenic bacterium. The insights from our work not only enhance our understanding of sRNA functions in bacterial virulence but also underscore the potential of sRNAs as targets for novel antimicrobial strategies. Further research is essential to reveal the complete spectrum of sRNA-mediated regulation and its impact on the pathogenicity of *P. aeruginosa*, particularly in clinical settings.

## CRediT authorship contribution statement

**Tianmin Li:** Writing – review & editing, Writing – original draft, Validation, Project administration, Methodology, Investigation. **Beifang Lu:** Writing – review & editing, Software, Methodology, Data curation. **Fang Chen:** Writing – review & editing, Methodology. **Letong Xu:** Software, Data curation. **Yizhou Zhang:** Validation, Methodology. **Fangzhou Xie:** Methodology, Data curation. **Yue Sun:** Writing – review & editing. **Chunyan Yao:** Conceptualization. **Jiadai Huang:** Writing – review & editing. **Tianlong He:** Methodology. **Youyue Li:** Methodology. **Yiqing Ding:** Writing – review & editing. **Haiyan Hu:** Writing – review & editing. **Shaojun Tang:** Writing – review & editing, Investigation. **Xin Deng:** Writing – review & editing, Supervision, Funding acquisition.

## Data availability

The RNA-seq data have been uploaded under the accession code GSE267905 in the National Center for Biotechnology Information (NCBI) Gene Expression Omnibus (GEO) database.

**Code availability**

The source code in this paper is publicly accessible on GitHub at https://github.com/dengxinb2315/CCEP-sRNA.

## Funding

This study was funded by the Guangdong Major Project of Basic and Applied Basic Research (China) (No. 2020B0301030005), 10.13039/501100004791Shenzhen Science and Technology Fund (China) (No. JCYJ20210324134000002), the 10.13039/501100001809National Natural Science Foundation of China (No. 32172358, 31670127, 31870116), Hong Kong 10.13039/100018696Health and Medical Research Fund (China) (No. 20190942), Hong Kong Research Grants Council Collaborative Research Fund (China) (No. C7033-20G), and General Research Fund (China) (No. 21103018, 11101619, 11102720). The funders had no role in the study design, data collection, interpretation, or decision to submit the work for publication.

## Conflict of interests

Xin Deng is a member of *Genes & Diseases* Editorial Board. To minimize bias, he/she was excluded from all editorial decision-making related to the acceptance of this article for publication. The remaining authors declare no conflict of interest.

## Figures and Tables

**Figure S1:**
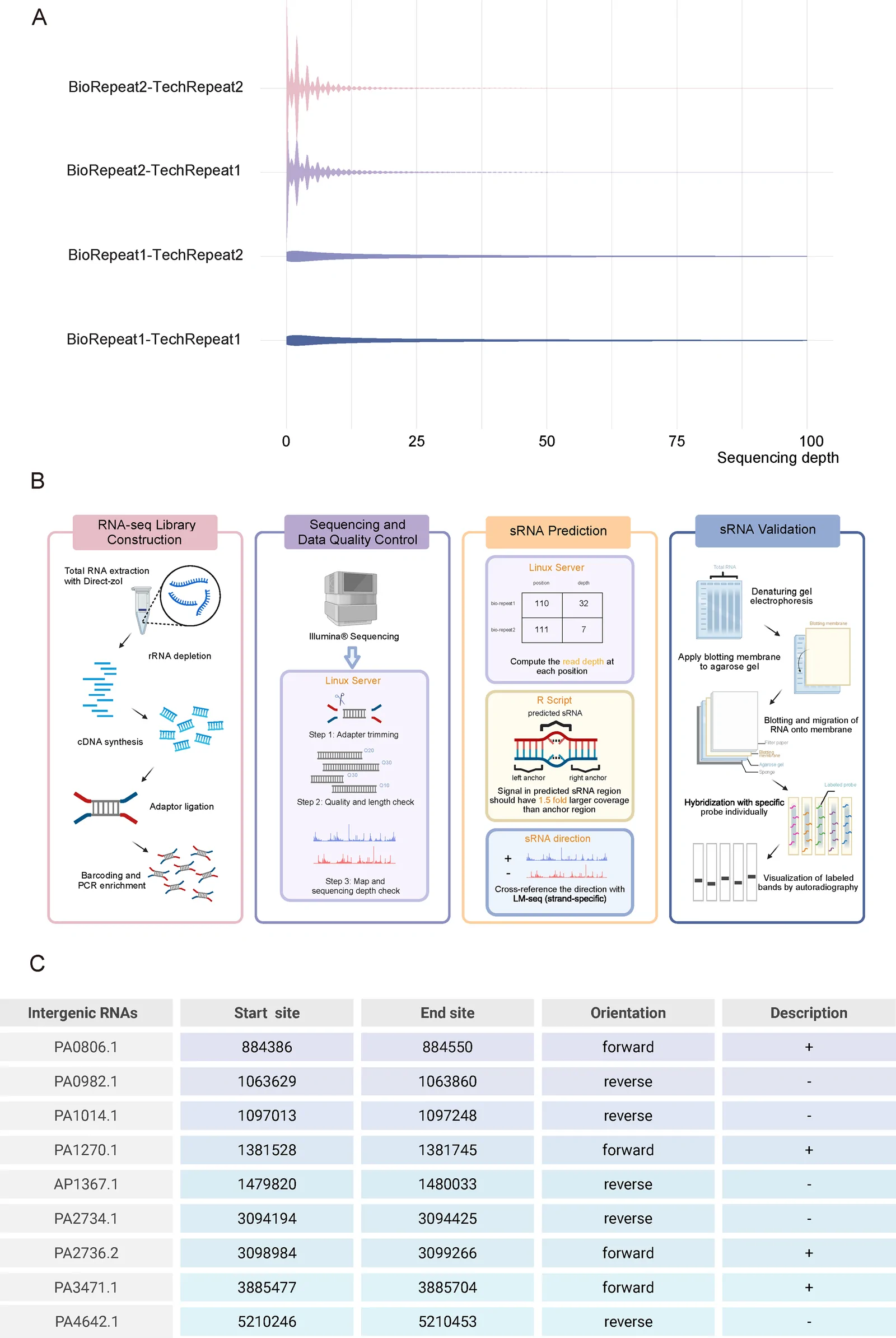
Optimization and validation for intergenic RNA identification. (A) Sequencing depth profiles of 4 RNA libraries generated from 2 biological replicates, each with 2 technical replicates. (B) Workflow diagram illustrating the steps of sRNA prediction and validation, including RNA-seq library construction, sequencing and data quality control, sRNA prediction, and experimental validation. (C) Genomic locations of predicted sRNA signals in *P. aeruginosa*. The table summarizes their chromosomal start and end coordinates and strand orientations.

**Figure S2:**
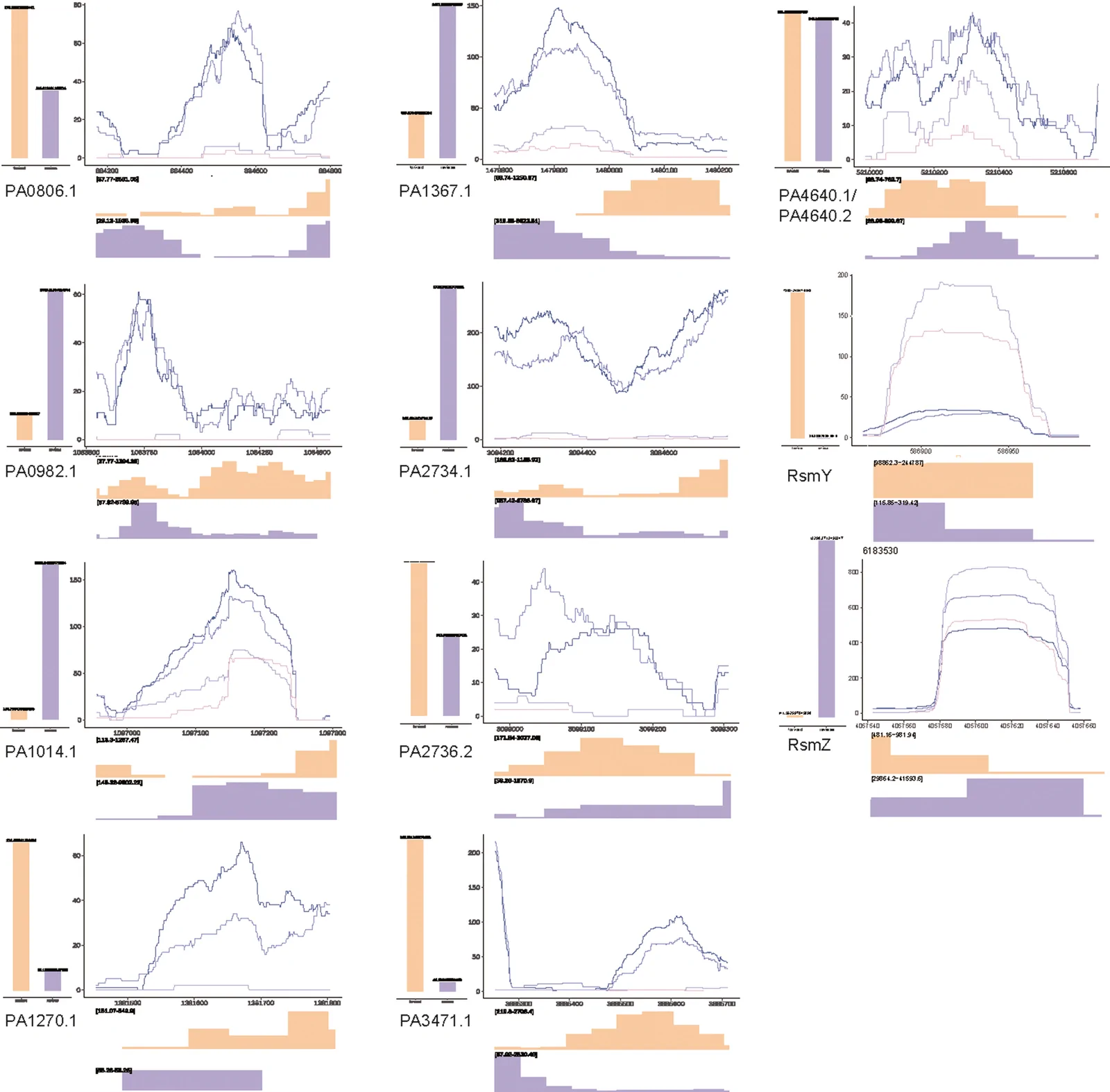
sRNA transcriptional orientations and basal expression level in PAO1. sRNA strand-specific mapping scores from LM-seq. The figure below the *x*-axis shows mapping scores along the genome with the coding strand in yellow and the minus strand in purple, demonstrating the distribution of sequence alignment across both strands. Adjacent to the *y*-axis, the bar chart summarizes the total mapping scores for each strand.

**Figure S3:**
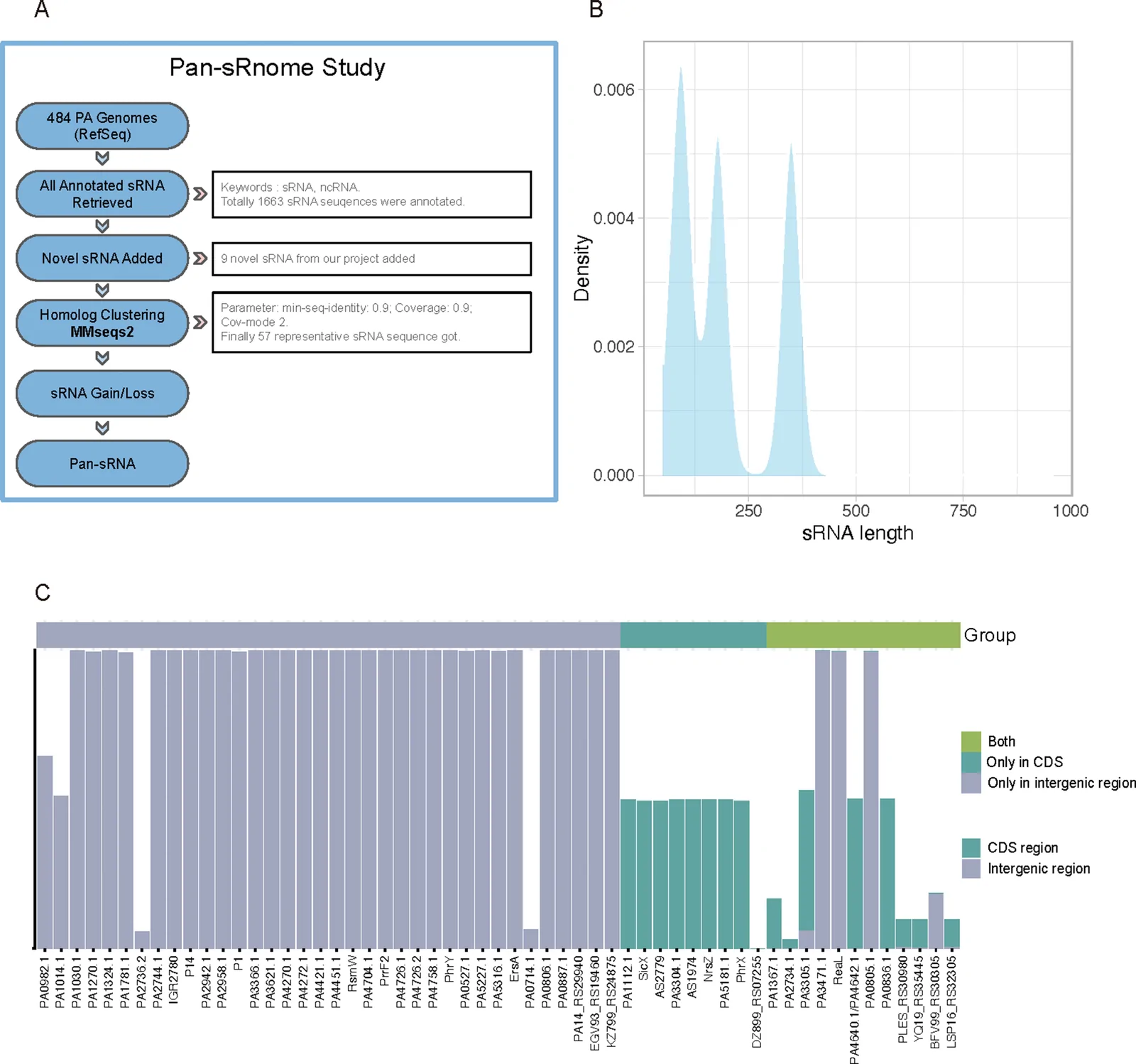
Workflow of Pan-sRNome study and characterization of 58 sRNA homologs in 484 *P. aeruginosa* strains. (A) Flowchart of defining pan-sRNome. (B). Density distribution of sRNA lengths identified in the *P. aeruginosa* Pan-sRNome. The kernel density plot illustrates the frequency of sRNAs across a range of transcript lengths. (C). sRNA genomic localization and abundance. The *x*-axis categorizes sRNA sequence homologs, and the *y*-axis quantifies their occurrence. Gray bars indicate sequences in IGR, dark green bars represent those located solely within the CDS region, and light green bars indicate sequences present in both CDSs and IGRs.

**Figure S4:**
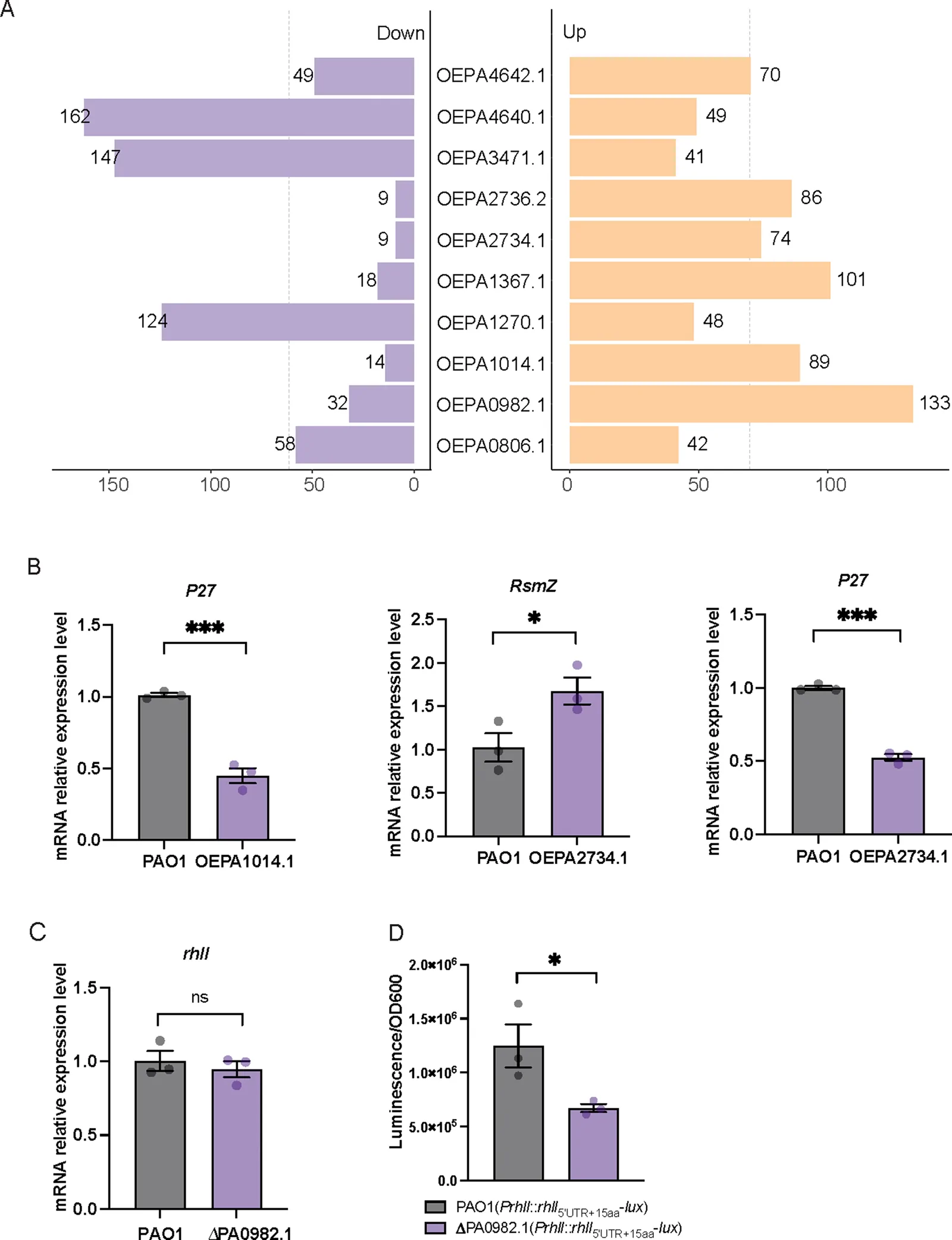
Differential gene expression induced by overexpression of 9 sRNAs in PAO1. (A) Purple bars represent the counts of downregulated genes, and yellow bars indicate the counts of upregulated genes across the 9 sRNA overexpression strains. (B) RT‒qPCR validation of sRNA–sRNA interactions (C) Relative mRNA expression levels of *rhlI* in PAO1 and *ΔPA0982.1* strains determined by RT‒qPCR (D) Analysis of *rhlI* expression using a translational reporter fusion. Data represent relative luminescence normalized to OD_600_.

**Figure S5:**
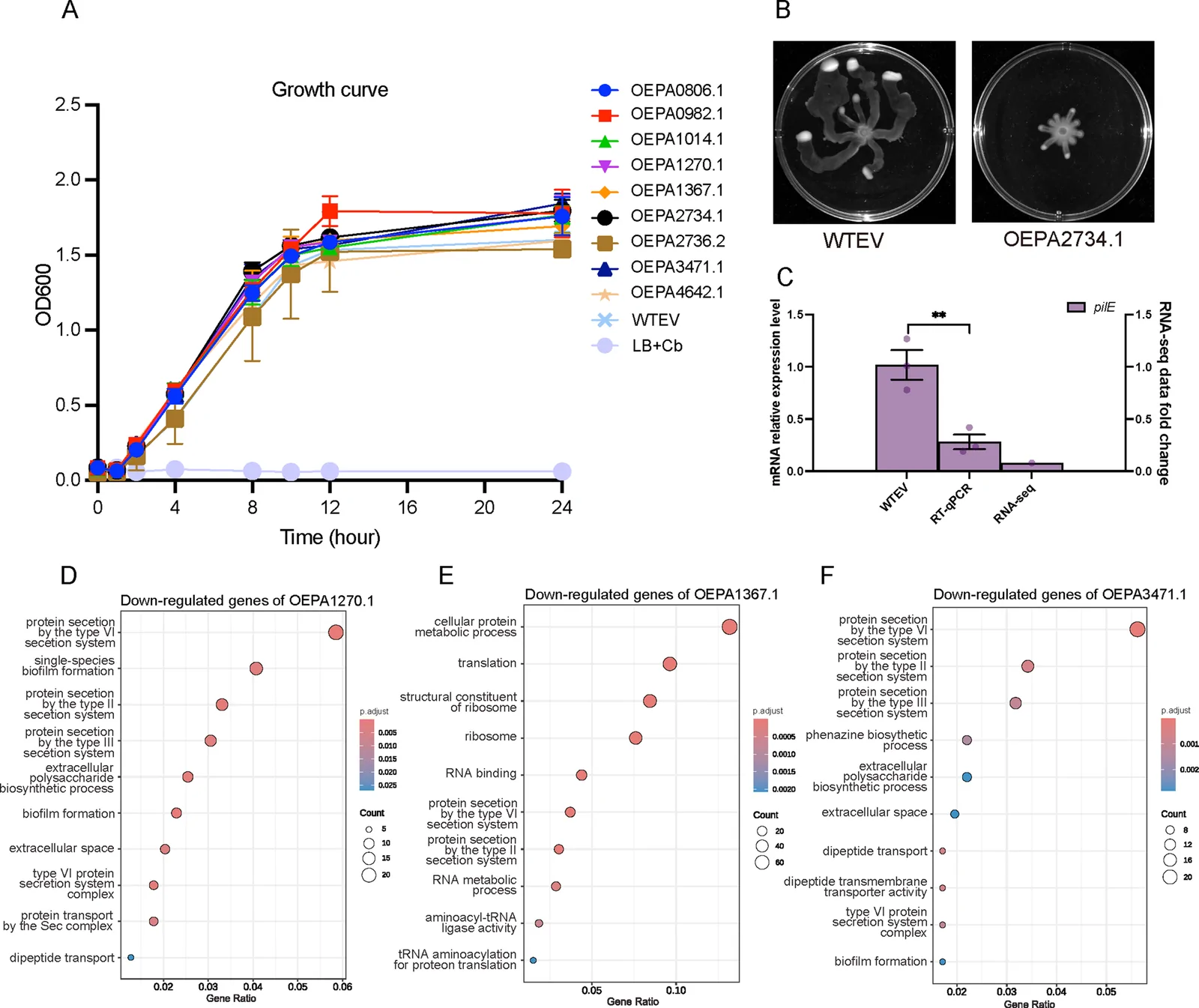
Impact of sRNA overexpression on virulence and bacterial competition. (A) Growth curves of sRNA overexpression strains compared to WTEV in LB. (B) Swarming motility detection of PA2734.1. (C) Detection of gene *pilE* expression in the WTEV and PA2734.1 overexpression strains by RT‒qPCR. (D–F) Bubble plot of enriched GO terms for downregulated DEGs comparing PA1270.1, PA1367.1, and PA3471.1 overexpression strains to WTEV.

**Figure S6:**
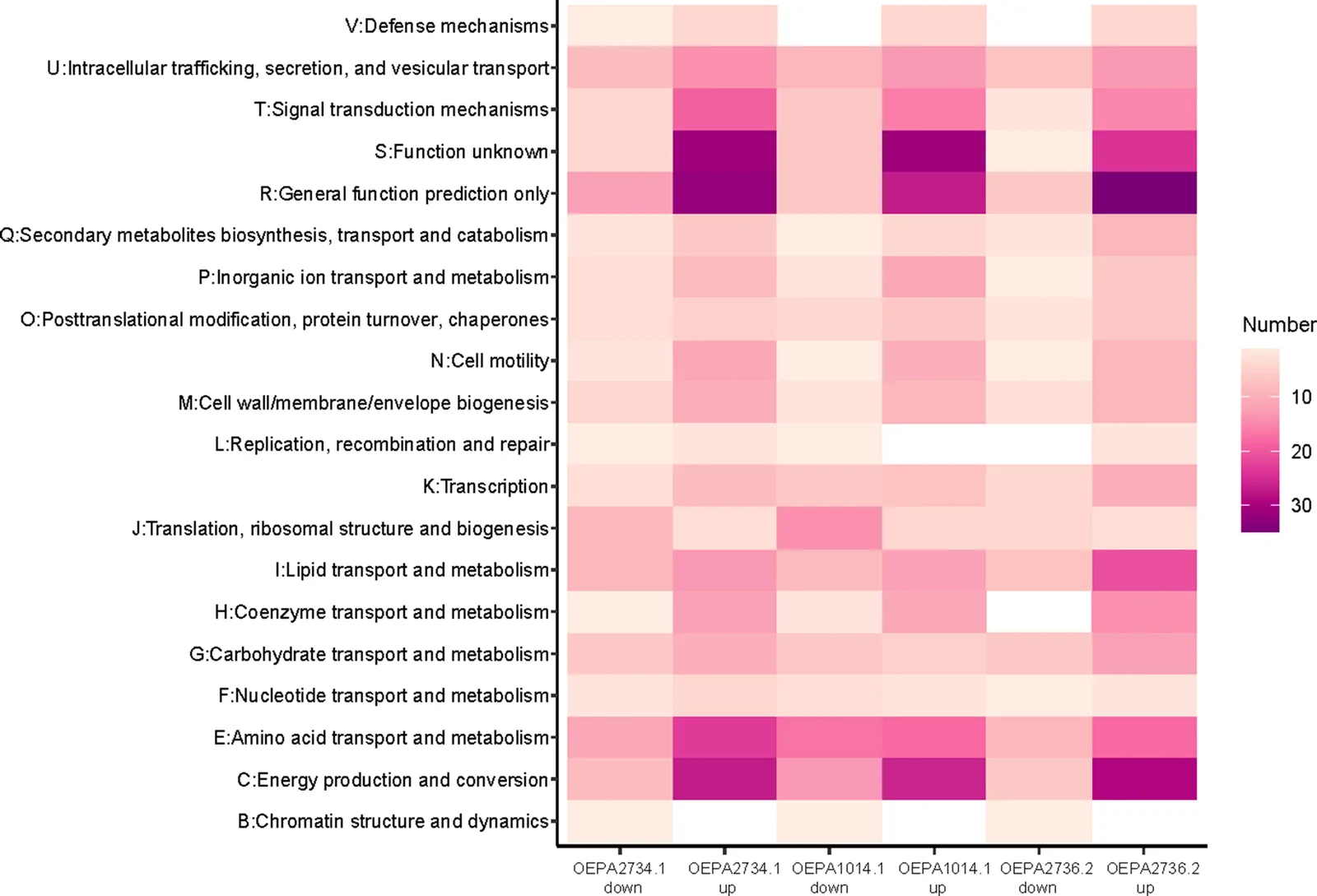
COG functional analysis of protein expression in the PA1014.1, PA2734.1, and PA2736.2 overexpression strains. The heatmap illustrates the differential expression of proteins in the 3 overexpression strains. The color intensity reflects the number of downregulated and upregulated proteins, categorized by COG functional groups, with the scale indicating the count of affected proteins.

**Figure S7:**
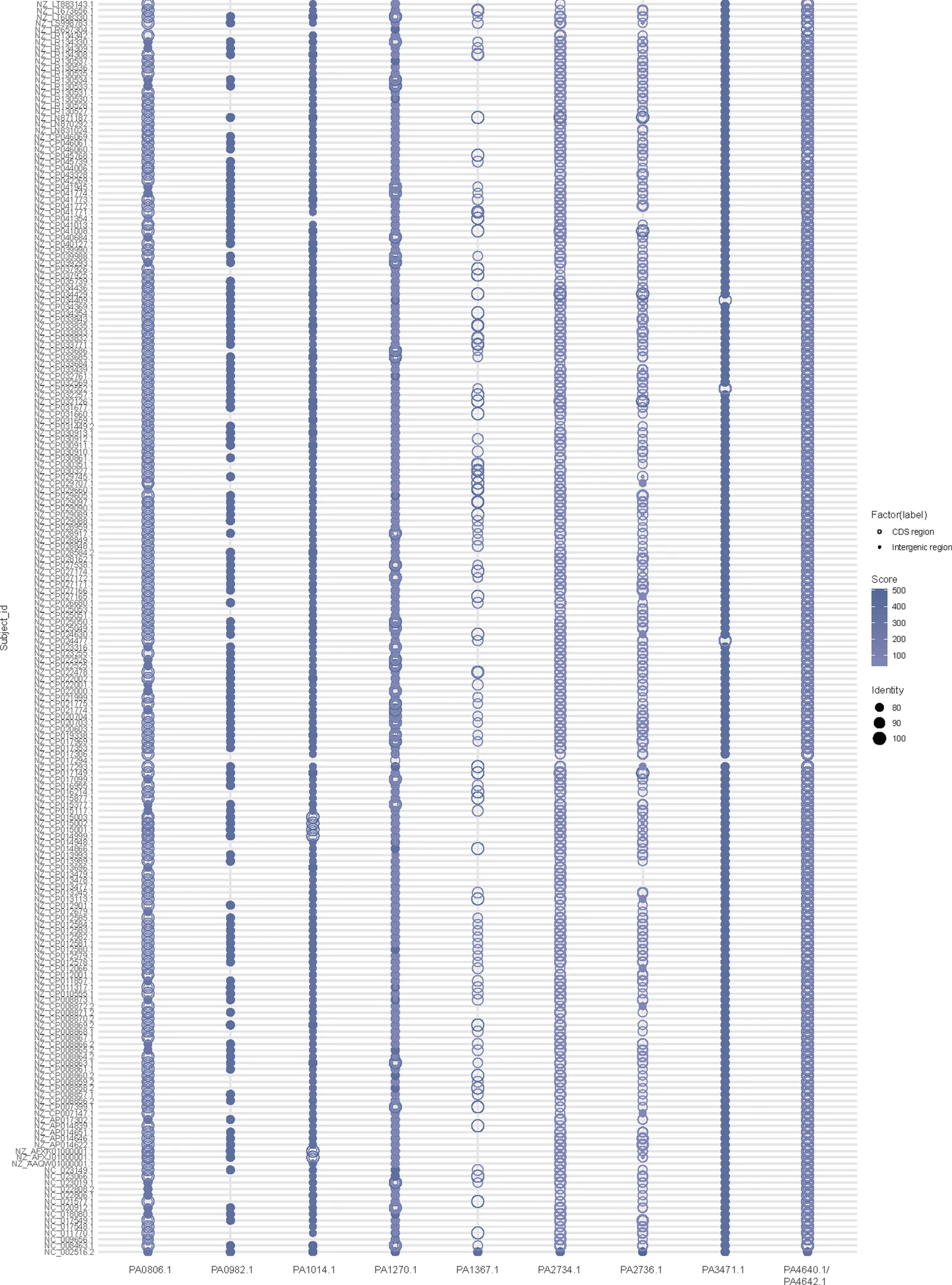
Phylogenetic analysis of sRNA homolog conservation across 200 *P. aeruginosa* strains. The dot plot illustrates the conservation of sRNA homologs across 200 *P. aeruginosa* strains, with rows representing individual sRNAs and columns representing the strain dataset. The color intensity reflects the sequence alignment score, and identity scores indicate intraspecies conservation.
