# Supplementary material for: Identification and functional analysis of sRNAs in Pseudomonas aeruginosa quorum sensing and virulence regulation
Source: Genes Dis. 2026 Jun 11;14(2):102292. doi: 10.1016/j.gendis.2026.102292 (PMC13677034; doi:10.1016/j.gendis.2026.102292)
Supplement: Supplementary file 1 [file mmc2.docx]

Table S2. Summary of phenotypic categories regulated by the nine newly identified sRNAs

| **sRNA** | **Quorum Sensing (QS)** | | **Pyocyanin (PYO)** | **Biofilm Formation** | **Motility** | **T6SS** |
| --- | --- | --- | --- | --- | --- | --- |
| PA0806.1 | |  |  |  |  |  |
| PA0982.1 | | + | + |  |  |  |
| PA1014.1 | | + | + | + |  |  |
| PA1270.1 | |  |  |  |  | - |
| PA1367.1 | |  |  |  |  | - |
| PA2734.1 | | + | + | + | - |  |
| PA2736.2 | | + | + |  | - |  |
| PA3471.1 | |  |  |  |  | - |
| PA4642.1 | |  |  |  |  |  |
